# A multicentre single arm phase 2 trial of neoadjuvant pyrotinib and letrozole plus dalpiciclib for triple-positive breast cancer

**DOI:** 10.1038/s41467-022-34838-w

**Published:** 2022-11-17

**Authors:** Nan Niu, Fang Qiu, Qianshi Xu, Guijin He, Xi Gu, Wenbin Guo, Dianlong Zhang, Zhigao Li, Yi Zhao, Yong Li, Ke Li, Hao Zhang, Peili Zhang, Yuanxi Huang, Gangling Zhang, Hongbin Han, Zhengang Cai, Pengfei Li, Hong Xu, Guanglei Chen, Jinqi Xue, Xiaofan Jiang, Alireza Hamidian Jahromi, Jinshi Li, Yu Zhao, Eduardo de Faria Castro Fleury, Shiwen Huo, Huajun Li, Guy Jerusalem, Domenico Tripodi, Tong Liu, Xinyu Zheng, Caigang Liu

**Affiliations:** 1 Department of Oncology, Shengjing Hospital of China Medical University, Shenyang, China; 2 Innovative Cancer Drug Research and Development Engineering Centre of Liaoning Province, Shenyang, China; 3 Department of Breast Surgery, Dalian Municipal Central Hospital, Affiliated Hospital of Dalian Medical University, Dalian, China; 4 Department of Breast Surgery, Affiliated Zhongshan Hospital of Dalian University, Dalian, China; 5 Department of Breast Surgery, Cancer Hospital of Harbin Medical University, Harbin, China; 6 Department of Breast Surgery, Benxi Central Hospital, The Fifth Affiliated Hospital of China Medical University, Benxi, China; 7 Department of Breast Surgery, Anshan Cancer Hospital, Anshan, China; 8 Department of Breast Surgery, Cancer Hospital of China Medical University, Liaoning Cancer Hospital and Institute, Shenyang, China; 9 Department of Breast Surgery, Baotou Cancer Hospital, Baotou, China; 10 Department of Breast Surgery, Liaohe Oilfield General Hospital, Affiliated Hospital of China Medical University, Panjin, China; 11 Department of Breast Surgery, the First Affiliated Hospital of Dalian Medical University, Dalian, China; 12 Department of Thoracic and Breast Surgery, Yan’an People’s Hospital, Yan’an, China; 13 Division of Plastic and Reconstructive Surgery, Temple University Medical Center, Philadelphia, PA USA; 14 Department of Anesthesiology, the First Affiliated Hospital of China Medical University, Shenyang, China; 15 Department of Biochemistry and Molecular Biology, Mayo Clinic College of Medicine, Rochester, MN 55905 USA; 16 Instituto Brasileiro de Controle do Câncer Oncologia (IBCC Oncologia), São Paulo, Brazil; 17 Jiangsu Hengrui Pharmaceuticals, Shanghai, China; 18 Medical Oncology Department, CHU Liège and Liège University, Liege, Belgium; 19 Department of Surgical Sciences, Sapienza University of Rome, Rome, Italy; 20 Department of Breast Surgery, The First Affiliated Hospital of China Medical University, Shenyang, China

**Keywords:** Breast cancer, Combination drug therapy

## Abstract

Current therapies for HER2-positive breast cancer have limited efficacy in patients with triple-positive breast cancer (TPBC). We conduct a multi-center single-arm phase 2 trial to test the efficacy and safety of an oral neoadjuvant therapy with pyrotinib, letrozole and dalpiciclib (a CDK4/6 inhibitor) in patients with treatment-naïve, stage II–III TPBC with a Karnofsky score of ≥70 (NCT04486911). The primary endpoint is the proportion of patients with pathological complete response (pCR) in the breast and axilla. The secondary endpoints include residual cancer burden (RCB)−0 or RCB-I, objective response rate (ORR), breast pCR (bpCR), safety and changes in molecular targets (Ki67) from baseline to surgery. Following 5 cycles of 4-week treatment, the results meet the primary endpoint with a pCR rate of 30.4% (24 of 79; 95% confidence interval (CI), 21.3–41.3). RCB-0/I is 55.7% (95% CI, 44.7–66.1). ORR is 87.4%, (95% CI, 78.1–93.2) and bpCR is 35.4% (95% CI, 25.8–46.5). The mean Ki67 expression reduces from 40.4% at baseline to 17.9% (*P* < 0.001) at time of surgery. The most frequent grade 3 or 4 adverse events are neutropenia, leukopenia, and diarrhoea. There is no serious adverse event- or treatment-related death. This fully oral, chemotherapy-free, triplet combined therapy has the potential to be an alternative neoadjuvant regimen for patients with TPBC.

## Abstract (web summary)

Neoadjuvant therapy is recommended for patients with locally advanced breast cancer. Here the authors report the results of a phase 2 clinical trial of oral neoadjuvant therapy with pyrotinib (pan-HER tyrosine kinase inhibitor), letrozole (aromatase inhibitor) and dalpiciclib (CDK4/6 inhibitor) in patients with treatment-naïve and stage II-III triple positive breast cancer.

## Introduction

Human epidermal growth factor receptor 2 (HER2) overexpression is found in approximately 15–20% of breast cancer patients with an aggressive phenotype and detrimental outcome^1^. More than 50% of HER2-positive breast cancers express intact estrogen receptor (ER) and progesterone receptor (PR), termed triple-positive breast cancer (TPBC)^2^. TPBC displays unique biological behaviors and manifests a poorer response to standard neoadjuvant therapy than hormone receptor (HR)-negative, HER2-positive breast cancer^3,4^. A retrospective study reported that neoadjuvant chemotherapy combined with trastuzumab treatment only resulted in a pathological complete response (pCR) rate of 20% in patients with TPBC, the lowest among all subgroups of HER2-positive breast cancers^5^, indicating that optimized therapeutic strategies are urgently required for the management of TPBC. However, few studies have focused on the treatment exploration for TPBC in the neoadjuvant setting.

Ample evidence has unveiled that complex crosstalk between HER2 and ER signaling lays the foundation for poor response to current therapies in patients with TPBC and for a rationale to block them simultaneously^6,7^. Clinical trials have shown that the combination of HER2-targeted therapy with endocrine therapy achieved a moderate therapeutic efficacy in HR-positive, HER2-positive breast cancer^8,9^. In the TBCRC 006 study, the pCR rate of neoadjuvant lapatinib and trastuzumab plus letrozole was 18% in patients with ER-positive, HER2-positive breast cancer^8^. In the TBCRC 023 study, patients with ER-positive, HER2-positive breast cancer receiving 12-week or 24-week treatment had a bpCR of 9% or 33% with minimal side effects^9^. However, the curative efficacy of this combinatory regimen remains unsatisfactory.

Given the multiple signaling-driven features, treatment of TPBC with multi-targeted agents may block the complex network more effectively. The HER2 and ER signals converge on RB1, and inhibition of cyclin D1 and cyclin-dependent kinase (CDK) 4 can reverse resistance to HER2-targeted therapy in HER2-positive breast cancer^10^. Furthermore, a higher *CCND1* amplification rate was also detected in TPBC compared with HR-negative, HER2-positive breast cancer, and more than 80% of TPBC were characterized by luminal A/B intrinsic subtype, indicating the sensitivity of TPBC to CDK4/6 inhibitor and endocrine therapy^2^. In the monarcHER study, the combination of CDK4/6 inhibitor abemaciclib, trastuzumab, and fulvestrant has shown a superior progression-free survival and a tolerable safety profile compared with trastuzumab plus standard chemotherapy in patients with heavily pretreated HR-positive, HER2-positive metastatic breast cancer^11^. However, evidence of this combination strategy for TPBC in the neoadjuvant setting is scarce.

Dalpiciclib (SHR6390) is a novel oral CDK4/6 inhibitor^12^, and pyrotinib is an oral irreversible pan-HER tyrosine kinase inhibitor^13^. Preclinical studies have suggested that dalpiciclib can overcome the resistance to HER2-targeted blockade and endocrine therapy in ER-positive, HER2-positive breast cancer cells^14^. The phase 1b LORDSHIPS study has suggested a potentially synergistic anti-tumor effect with pyrotinib, letrozole, and dalpiciclib in front-line patients with HR-positive, HER2-positive metastatic breast cancer^15^.

In this work, we conduct the MUKDEN 01 study to investigate the efficacy and safety of neoadjuvant therapy with pyrotinib and letrozole plus dalpiciclib in TPBC patients to explore the treatment strategy for TPBC. In addition, these orally administered therapeutics may be beneficial for patient compliance and ensure therapeutic continuity under challenging treatment conditions.

## Results

### Patient characteristics

**Fig. 1:**
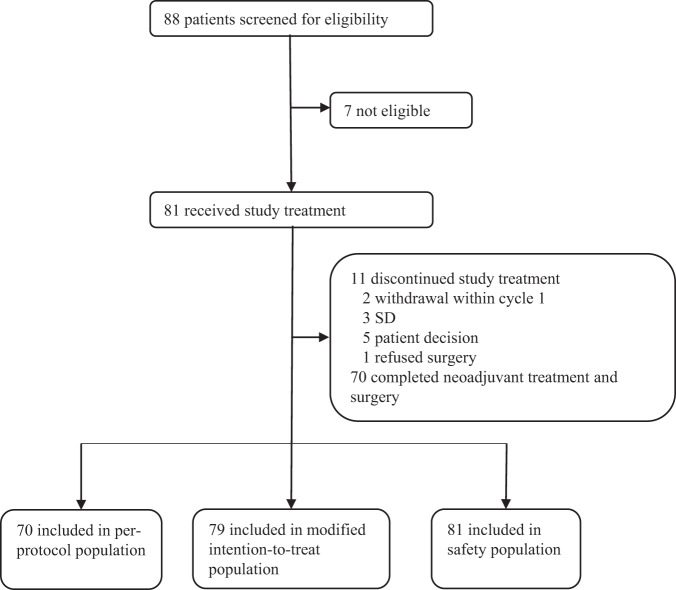
Trial profile. Treatment summary and data collection of study participants.

Between July 27, 2020, and May 13, 2022, a total of 88 patients were screened for eligibility, and 81 patients were recruited to receive 5 cycles of 4-week neoadjuvant treatment (Fig. 1). Among these 81 patients, 2 withdrew the informed consent during cycle 1, thus 79 were included in the modified intention-to-treat (mITT) population and 81 in the safety population. The baseline characteristics are shown in Table 1. There were 68% of patients with stage IIB-III TPBC at the time of diagnosis and 68% with lymph node metastases (N1 or above). The mean percentages of ER- and PR-expressing tumor cells in core-needle biopsied tissue samples were 78 and 49%, respectively.

**Table 1:** Baseline and disease characteristics of patients

	Patients (*n* = 81)
*Median age, years (range)*	51 (26–75)
<55	49 (60%)
≥55	32 (40%)
*Menopausal status*	
Pre- or perimenopausal	43 (53%)
Postmenopausal	38 (47%)
*Stage at baseline*	
IIA	26 (32%)
IIB	26 (32%)
III	29 (36%)
*Nodal status*	
N0	26 (32%)
N1	30 (37%)
N2	21 (26%)
N3	4 (5%)
*Tumor size*	
T1	4 (5%)
T2	70 (86%)
T3-4	7 (9%)
ER expression, mean (SD), %	78% (18)
PR expression, mean (SD), %	49% (31)
Ki67 level at baseline, mean (SD), %	40% (20)
*HER2 status*	
IHC 2+/FISH+	25 (31%)
IHC 3+	56 (69%)

Data are *n* (%) or mean (SD).

*SD* standard deviation, *ER* estrogen receptor, *PR* progesterone receptor.

*IHC*, immunohistochemistry, *FISH*, Fluorescence in situ hybridization.

### Pathological and clinical response

**Fig. 2:**
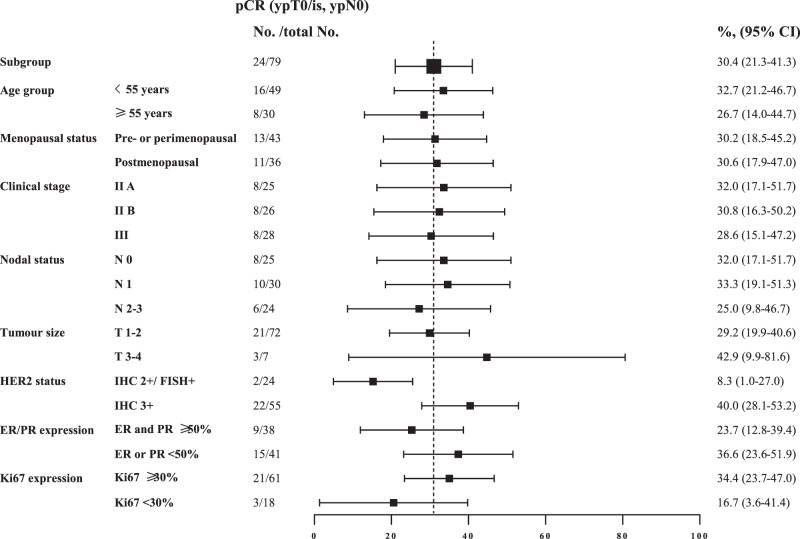
Exploratory subgroup analyses of pCR by baseline factors. Data are presented as pCR rate (%) and 95% CI. The black squares indicate the pCR rates following stratifications. *pCR* pathological complete response, *HER2* human epidermal growth factor receptor 2, *IHC* immunohistochemistry, *FISH* fluorescence in situ hybridization, *ER* estrogen receptor, *PR* progesterone receptor, *CI* confidence interval. Source data are provided as a Source Data file.

According to the study protocol, 61 cases were enrolled in the first stage, and following the combined therapy, 18 cases (29.5%, 95% CI, 18.5–42.6) achieved pCR, meeting the minimum number of responders required to continue the study. In the overall mITT population, analysis of the resected breast tissues and lymph nodes found that this combined therapy resulted in 24 (30.4%, 95% CI, 21.3–41.3) of 79 patients achieving pCR in both the breast and lymph nodes and 28 (35.4%, 95% CI, 25.8–46.5) of 79 patients achieved pCR in the breast only (Table 2). Similar results were observed in the per-protocol (PP) population (*n* = 70) with a pCR rate of 31.4% (95% CI, 21.7–43.1) and bpCR rate of 37.1% (95% CI, 26.8–48.9) (Table 2). The proportion of patients with residual cancer burden (RCB)−0 or RCB-I was 55.7% (95% CI, 44.7–66.1) in the mITT population and 60.0% (95% CI, 48.3–70.7) in the PP population (Table 2). A post-hoc analysis unveiled that pCR tended to be higher in patients with HER2 3+ by immunohistochemistry (40.0%, 95% CI, 28.1–53.2) than those with HER2 2 + (8.3%, 95% CI, 1.0–27.0) (Fig. 2). Regarding the radiographic response to neoadjuvant therapy, 69 (87.4%, 95% CI, 78.1–93.2) patients achieved objective response before surgery (Table 2).

**Table 2:** Pathological and clinical responses

	Modified intention-to-treat population	Per-protocol population
	(*n* = 79)	(*n* = 70)
Total pathological complete response	24 (30%)	22 (31%)
Pathological complete response in breast	28 (35%)	26 (37%)
Residual cancer burden score		
0	24 (30%)	22 (31%)
I	20 (25%)	20 (29%)
II	21 (27%)	18 (26%)
III	14 (18%)	10 (14%)
Complete response	4 (5%)	4 (6%)
Partial response	65 (82%)	59 (84%)
Stable disease	10 (13%)	7 (10%)

Data are n (%).

### Ki-67 expression at baseline and surgery

**Fig. 3:**
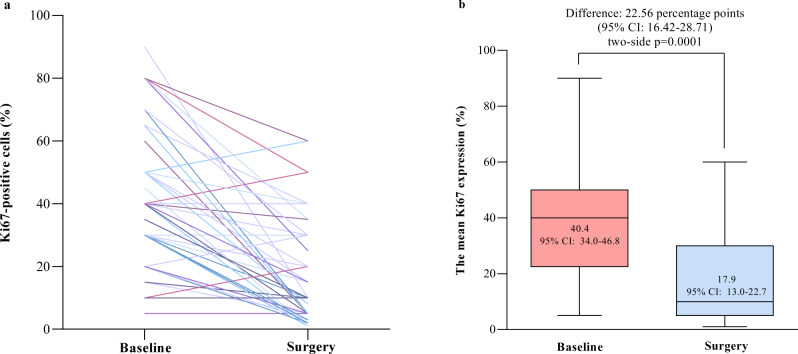
Ki-67 expression at baseline and surgery. **a** Individual data (*n* = 48). **b** Mean percentages of Ki-67 expressing tumor cells of each group (*n* = 48). Statistical analysis was performed by paired two-sided *t*-test. The Ki-67 expression in 31 out of 79 patients was not assessable due to the lack of detectable tumor cells in the surgical samples (*n* = 30) or not undergoing surgery (*n* = 1). For box plots, the center lines are group medians; the upper and lower limits of the box are the 1st and 3rd quartile, and the whiskers show minimum and maximum values. *CI* confidence interval. Source data are provided as a Source Data file.

Analyses of 48 baselines biopsied and resected breast samples indicated that the mean Ki67 positive rate reduced from 40.4% (95% CI, 34.0–46.8) at baseline to 17.9% (95% CI, 13.0–22.7; *P* < 0.001) in the surgical samples (Fig. 3a, b). There were 31 patients without Ki67 data due to the lack of detectable tumor cells in their surgical samples (*n* = 30) or not undergoing surgery (*n* = 1) in this population.

### Safety

In the safety population, all 81 patients experienced at least one adverse event. The most common adverse events were neutropenia (75 [93%]), leukopenia (73 [90%]), diarrhea (70 [86%]), anemia (55 [68%]), and oral mucositis (51 [63%]), which occurred in ≥50% of 81 patients (Table 3). Among those patients, 55 (68%) patients experienced grade 3 or 4 adverse events, with the most common being neutropenia (43 [53%]), leukopenia (16 [20%]), and diarrhea (14 [17%]). Six reversible grade 4 toxicities were observed in the study. Specifically, a grade 4 aspartate aminotransferase increased event was observed in one patient, and grade 4 neutropenia appeared in five patients, all of which normalized after a dose interruption. There were 17 (21%) patients with dose interruptions for pyrotinib (seven, 9%), dalpiciclib (one, 1%), or both (nine, 11%), respectively. Two (2%) patients required a dose reduction in dalpiciclib to 100 mg because of grade 3 neutropenia (*n* = 1) and grade 3 oral mucositis (*n* = 1). There was no patient, who discontinued study treatment due to adverse events. There were no serious adverse events or treatment-related deaths in this population.

**Table 3:** Treatment-emergent adverse events in patients (*n* = 81)

	No. (%)		
	Grade 1 or 2	Grade 3	Grade 4
Neutropenia	32 (40%)	38 (47%)	5 (6%)
Leukopenia	57 (70%)	16 (20%)	0
Diarrhea	56(69%)	14 (17%)	0
Anemia	52 (64%)	3 (4%)	0
Oral mucositis	47 (58%)	4 (5%)	0
Nausea	26 (32%)	0	0
Rash	22 (27%)	1 (1%)	0
Hypokalemia	20 (25%)	1 (1%)	0
*γ-glutamyl transferase*			
Increased	15 (19%)	1 (1%)	0
Fatigue	13 (16%)	1 (1%)	0
Hyponatremia	13 (16%)	0	0
Vomiting	13 (16%)	0	0
Headache	11 (14%)	0	0
Hypocalcemia	10 (12%)	0	0
Platelet count decreased	9 (11%)	0	0
Dizziness	9 (11%)	0	0
Hypercholesteremia	7 (9%)	1 (1%)	0
Alopecia	5 (6%)	0	0
*Alanine aminotransferase*			
Increased	2 (2%)	2 (2%)	0
*Aspartate aminotransferase*			
Increased	0	0	1 (1%)

Data are presented as n (%).

### Patient-reported outcome

**Fig. 4:**
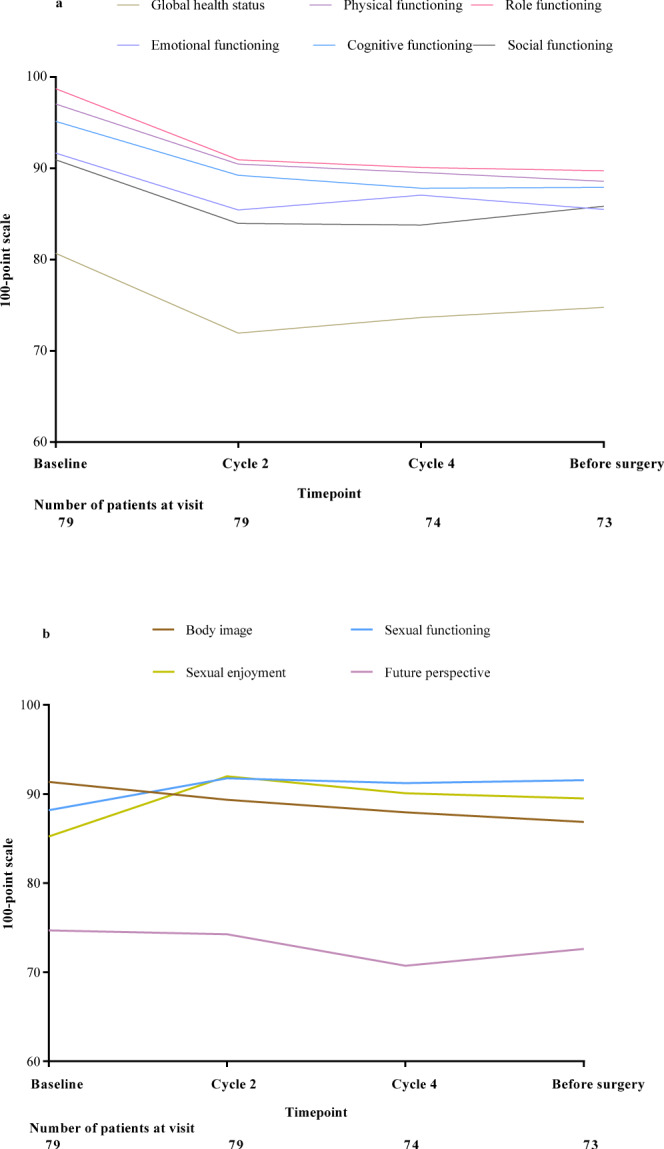
Results of health-related quality of life questionnaires in QLQ-C30 over time. **a** Mean scores of global health status and functioning subscales in QLQ-C30. **b** Mean scores of functioning subscales in QLQ-BR23. *QLQ-C30* quality of life questionnaire-core module, *QLQ-BR23* quality of life questionnaire-breast cancer module. Source data are provided as a Source Data file.

We further assessed the quality of life as an explorative objective. There were 79 patients with available results from the Quality of Life questionnaire. The global health status and functioning subscales in QLQ-C30 tended to be reduced in the first two months post-administration, but remained stable thereafter (Fig. 4a). The mean physical functioning subscales in QLQ-BR23 remained stable throughout the treatment period (Fig. 4b). Mean change from baseline at any time point in neither questionnaire exceeded the threshold for a clinically meaningful deterioration.

## Discussion

The MUKDEN 01 trial evaluated the therapeutic efficacy and safety of neoadjuvant pyrotinib and letrozole plus dalpiciclib for TPBC, to explore the potential of a fully oral, chemotherapy-free neoadjuvant regimen. This combinational therapy exhibited a promising anti-tumor activity with a pCR rate of 30.4% and an acceptable safety profile. The results indicate that this combinational therapy may be an alternative neoadjuvant regimen for patients with TPBC. Furthermore, fully oral administration may be more convenient for patients, especially in a specific condition, like the pandemic of COVID-19.

It is well established that patients with HER2-positive breast cancer have an inherent difference in response to the standard therapies between HR-positive and HR-negative subpopulations^2^. NeoSphere and PEONY studies found that the pCR rates in the HR-positive, HER2-positive subgroup were almost half of that in HR-negative, HER2-positive subgroup (NeoSphere: 22% vs. 55%; PEONY: 33% vs. 46%) following treatment with pertuzumab and trastuzumab plus docetaxel^4,16^. Though T-DXd has made a breakthrough for HER2-positive advanced breast cancer^17^, its efficacy in early-stage breast cancer is warranted to be explored.

A combination of HER2-targeted drug and endocrine therapy was conceptually feasible to optimize neoadjuvant therapy in patients with HR-positive, HER2-positive breast cancer because of the crosstalk between the ER and HER2 signaling. The TBCRC 006 and TBCRC 023 trials indicated that neoadjuvant therapy with lapatinib, trastuzumab, and letrozole resulted in a modest efficacy (TBCRC 006: pCR of 18% following 12-week therapy; TBCRC023: bpCR of 9% or 33% following 12-week or 24-week therapy, respectively) in patients with ER-positive, HER2-positive breast cancer^8,9^. These data provided proof in principle that the chemotherapy-free therapeutic options were reasonable for ER-positive, HER2-positive breast cancer. However, there are few neoadjuvant studies specifically on TPBC. Given the multiple signaling-driven features, inhibition of multiple signaling pathways may rationalize neoadjuvant therapy to improve efficacy in patients with TPBC.

The cyclin D1/CDK4 axis mediates the resistance to trastuzumab in HER2-positive breast cancer models, which can be reverted by CDK4/6 inhibition^10^, providing a solid rationale for simultaneous targeting HER2, ER, and CDK4/6 in HR-positive, HER2-positive breast cancer. In NA-PHER2, a four-drug regimen of neoadjuvant trastuzumab, pertuzumab, palbociclib, and fulvestrant led to a pCR rate of 27% and a rapid decrease in the frequency of Ki67-positive cells from baseline to week 2 in ER-positive, HER2-positive breast cancer^18^. Owing to a lower level of both HER2 expression and phosphorylation in TPBCs than those in HR-negative, HER2-positive breast cancers^2^, only one (2.0%) out of 51 patients achieved pCR with trastuzumab and pertuzumab in the HR-positive subgroup in the NeoSphere study^4^. It is reasonable to infer that palbociclib and fulvestrant may contribute to the increased pCR in the NA-PHER2 study. In monarcHER, similar progression-free survival was obtained from trastuzumab plus abemaciclib treatment and standard chemotherapy, indicating the importance of CDK4/6 inhibitor in the efficacy of the combined therapy for patients with HR-positive, HER2-positive breast cancer^11^.

Due to the limited efficacy of trastuzumab and pertuzumab in the HR-positive HER2-positive breast cancer subgroup, our study explored the activity and safety of the combinational therapy with oral HER2 tyrosine kinase inhibitor of pyrotinib, letrozole, and CDK4/6 inhibitor dalpiciclib in patients with stage II–III TPBC. This combination resulted in a pCR rate of 30.4%, numerically higher than that with pertuzumab and trastuzumab plus docetaxel in NeoSphere (22%)^4^, and comparable to that with the four-drug regimen in NA-PHER2 (27%)^18^. Given that pCR is not significantly associated with the prognosis of HR-positive, HER2-positive breast cancer, we further measured RCB as it is a prognostic marker for TPBC^19,20^. Notably, 55.7% of patients in our study had RCB-0 or RCB-I, suggesting that the combination with pyrotinib, letrozole, and dalpiciclib may lead to a favorable survival outcome in patients with TPBC.

Ki-67 is a common clinical measure of tumor cell proliferation^21^. It was found a significant decrease in the frequency of Ki-67 positive tumor cells at surgery after 5 cycles of 4-week combinational therapy (40.4% vs. 17.9%, baseline vs. surgery; *P* < 0.001), suggesting the antiproliferative effect of the triple-combination regimen in this population. It is notable that the combination of CDK4/6 inhibitor and endocrine therapy has led to a greater decrease in the frequency of Ki-67 positive tumor cells in HR-positive, and HER2-negative breast cancer^22–24^. However, a less pronounced reduction in the percentages of Ki-67-positive tumor cells was also observed in ER-positive, HER2-positive breast cancer following therapy with the four-drug regimen of trastuzumab, pertuzumab, palbociclib, and fulvestrant^18^. These may reflect the role of the HER2 pathway in the resistance of TPBC to the combined therapy.

Treatment-related adverse events and co-morbidities affect the patient’s quality of life and are crucial for the decision of treatment, especially for elderly patients and patients at a childbearing age^25,26^. We found that combinational therapy with pyrotinib, letrozole, and dalpiciclib was relatively safe with a good tolerability profile, as there was no serious adverse event or treatment-related death in this population. The treatment-emergent adverse events were as expected. Neutropenia, leukopenia, and diarrhea emerged as the most frequent grade 3 or 4 adverse events, which were easily manageable. It is worth mentioning that these adverse events occurring in our study were less severe than chemotherapy-related adverse events, such as alopecia, vomiting, hepatic functional abnormalities, and fatigue^27,28^. Our results suggest that combinational therapy with pyrotinib, letrozole, and dalpiciclib may be valuable for patients with TPBC, particularly for those who are unwilling to or unsuitable for chemotherapy. Furthermore, neoadjuvant chemotherapy usually requires the installation of central or peripheral venous catheters, which increases patients’ hospital visits and directly influences their quality of life. Conversely, the fully oral neoadjuvant regimen with pyrotinib, letrozole, and dalpiciclib provides a convenient alternative option for the treatment of patients with TPBC.

We recognized that our study had limitations, including the relatively small sample size, lack of a control group, and short-term follow-up, which may limit the interpretability of these results. In addition, we did not perform the 50-gene prediction analysis of microarray (PAM50) and obtain complete biomarker data in those patients due to the limited financial resource and biopsied samples as well as the unavailability of some specific reagents during the COVID-19 pandemic. We recognized that dual HER2-targeted blockades should have better benefits for HER2-positive breast cancer patients. In this study, because TPBC patients only received a single HER2 blockade drug, some patients without reaching pCR might be due to insufficient treatment with HER2 blockade and/or developing drug resistance, which may result in difficulty in selecting optimal adjuvant therapies after surgery. We have already planned a trial to test the therapeutic efficacy and safety of the combination of trastuzumab and current triplet drugs in TPBC patients to explore an enhanced curative efficacy (NCT05228951). Hence, treatment with dual HER2 blockades, particularly with anti-HER2 antibody, together with letrozole and dalpiciclib, as we designed in the new clinical trial, may achieve better therapeutic efficacy and help in selecting optimal adjuvant therapies for TPBC patients.

In conclusion, the neoadjuvant therapy with pyrotinib, letrozole, and dalpiciclib was well-tolerated and led to a promising pathological response in patients with TPBC. This fully oral, chemotherapy-free combinational neoadjuvant therapy has the potential to be an alternative neoadjuvant regimen for patients with TPBC. Further validation of these findings in a large-scale randomized controlled trial is warranted.

## Methods

The trial was designed and conducted, in accordance with the Declaration of Helsinki and the Guidelines for Good Clinical Practice (ClinicalTrials.gov identifier: NCT04486911). The trial protocol was approved by the Medical Ethics Committees of Shengjing Hospital of China Medical University, Baotou Cancer Hospital, Liaohe Oilfield General Hospital, and Yan’an People’s Hospital, and the Ethics Committee of Cancer Hospital of Harbin Medical University. Written informed consent was obtained from all participants before enrollment.

### Trial registration

This trial is registered under the name ‘Pyrotinib Maleate, CDK4/6 Inhibitor and Letrozole in Combination for Stage II-III TPBC: a Phase II Trial’ (ClinicalTrials.gov identifier: NCT04486911) on July 27, 2020.

### Study design and participants

The MUKDEN 01 was an investigator-initiated, multicentre, single-arm, phase 2 trial done at twelve hospitals in China. The trial began to enroll patients on July 27, 2020, and ended on May 13, 2022. All participants did not receive specific compensation, except for free-charged dalpiciclib for treatment during the trial. The inclusion criteria included: women aged 18–80 years; histologically confirmed stage II-III TPBC; treatment-naïve; Karnofsky score ≥ 70; and adequate bone marrow and organ function. TPBC was defined as HER2-positive (3+ by immunohistochemistry, or 2+ with fluorescence in situ hybridization positivity), ER-positive (more than 10% of tumor cells expressing estrogen receptor by immunohistochemistry), and PR-positive (at least 1% of tumor cells expressing progesterone receptor by immunohistochemistry) breast cancer.

The key exclusion criteria were: bilateral breast cancer, inflammatory breast cancer, or occult breast cancer; other malignancies within five years; serious comorbidities (i.e., uncontrolled hypertension, diabetes mellitus, or active infection); pregnancy, or lactating women. The trial protocol is available in the Supplementary Information.

### Procedures

Patients were treated with five cycles of 4-week neoadjuvant therapy. Each cycle consisted of pyrotinib 320 mg and letrozole 2.5 mg once daily for 28 days and dalpiciclib 125 mg once daily for 21 days, followed by 7 days off. Premenopausal or perimenopausal patients also received a subcutaneous injection with goserelin 3.6 mg on day 1 of each cycle for five cycles. Surgery was performed within 2–4 weeks after the last neoadjuvant therapy. Recommended surgery and adjuvant therapy followed the guideline of the National Comprehensive Cancer Network: radiotherapy (if indicated), endocrine treatment, postoperative chemotherapy and HER2 targeted therapy, including trastuzumab, pertuzumab, trastuzumab emtansine (T-DM1), or HER2 tyrosine kinase inhibitor. The necessity of additional chemotherapy for patients with pCR was judged by investigators.

The resected breast tissues were evaluated for pathological response and residual cancer burden (RCB). The RCB in individual samples was categorized as RCB-0 (no residual disease), RCB-I (minimal residual disease), RCB-II (moderate residual disease), and RCB-III (extensive residual disease)^29^. Ki-67 expression was determined by immunohistochemistry using a Ki67-specific rabbit monoclonal antibody (Ventana, catalog number H36867) assessed at baseline using biopsied tumor samples and after neoadjuvant therapy using resected tissues. A baseline MRI was performed within 4 weeks prior to the initiation of neoadjuvant therapy. Patients with a contraindication to MRI were assessed by breast ultrasound. The radiographic response to the neoadjuvant therapy was evaluated after the second and the fourth cycles of neoadjuvant therapy, and before surgery, according to the Response Evaluation Criteria in Solid Tumors (RECIST; version 1.1).

Adverse events were monitored from the day, when the subject signed the written informed consent, to 28 days after the last neoadjuvant therapy, according to the National Cancer Institute Common Terminology Criteria for Adverse Events version 5.0. Adverse events and tolerability were assessed by scheduled physical examinations, electrocardiograms, laboratory tests, and incidental testing.

Health-related quality of life (HRQoL) was measured using the European Organization for Research and Treatment of Cancer (EORTC) Quality of Life Questionnaire Core-30 (QLQ-C30; version 3) and Breast Cancer-Specific Module (QLQ-BR23): at baseline, at the end of cycles 2 and 4, and before surgery. The Patient-Reported Outcome (PRO) data were scored, according to the EORTC scoring manual^30^. A raw score was calculated as the average of items contributing to a scale and standardized to a range of 0–100 points. Higher scores on global health status and functioning subscales indicated better status. Higher scores on symptom subscales indicated worse symptoms.

### Study endpoints

The primary endpoint was the rate of pCR (ypT0/is, ypN0). The pCR (ypT0/is, ypN0) was defined as the disappearance of all invasive tumors in the breast and axillary lymph nodes. Patients, who did not undergo surgery, were regarded as not achieving the primary endpoint of pCR.

Secondary endpoints were the rate of breast pCR (bpCR, defined as the disappearance of all invasive tumors in the breast, ypT0/is), the proportion of patients with RCB-0 or RCB-I, change in Ki67 level from baseline to surgery (defined as the percentage of positively staining cells within the invasive margin in the examined area), objective response rate (proportion of patients with complete or partial response per RECIST 1.1) at the end of neoadjuvant treatment before surgery, and safety. Exploratory endpoints were changes in scores of different HRQoL dimensions from baseline to each time point.

### Deviations disclosed

With the exception of the Ki67 data, changes in molecular targets from baseline to surgery as secondary outcome pre-defined in the study protocol were not analyzed, including apoptosis marker, indicators related to CDK4/6, and indicators related to the pathways of CDK4/6 and HER-2 due to the limited financial resource.

### Statistical analyses

We calculated the sample size based on Simon’s minimax two-stage design. The null hypothesis of pCR rate was 15% and the alternative hypothesis was 26%, with a two-sided *α* of 0.05 and power of 80%. In the first stage, if 12 or more out of 61 patients achieved pCR, recruitment would proceed for additional 20 patients. If 18 or more out of 81 patients achieved pCR, the study treatment would be deemed worthy of future study.

The therapeutic efficacy was analyzed in the mITT and PP populations, respectively. The mITT population included patients, who received at least one cycle of neoadjuvant therapy. The PP population included patients, who completed five cycles of the neoadjuvant therapy and surgery without major violations. The safety of this neoadjuvant therapy was analyzed in the safety population, which included all patients, who received at least one dose of the study drugs and had available safety data.

Excel 2013 was used for data collection. Continuous data are presented as mean and standard deviation, or mean and 95% confidence interval. Categorical data are expressed as frequency and percentage. The 95% CIs of pCR rate, the proportion of patients with RCB-0 or RCB-I, and objective response rate were estimated using the Clopper–Pearson method. The difference in the mean percentages of Ki-67 expressing tumor cells between baseline and surgical samples was analyzed by paired *t*-test. Statistical analyses were performed using SAS (version 9.4).

### Reporting summary

Further information on research design is available in the Nature Portfolio Reporting Summary linked to this article.

## Supplementary information

Supplementary Information

Reporting Summary

## Data Availability

The raw clinical and imaging data are protected due to patient privacy laws. The datasets generated and/or analyzed during the current study are available from the corresponding author Caigang Liu on request for 10 years; de-identified clinical data and experimental data are available on request sharing, which will need the approval of the Institutional Ethical Committees. De-identified data will then be transferred to the inquiring investigator over secure file transfer. The study protocol is available as Supplementary Note 1 in the Supplementary Information. The remaining data are available within the Article, Supplementary Information or Source Data file. Source data are provided in this paper.

## Source data

Source Data
