# Supplementary material for: A multicentre single arm phase 2 trial of neoadjuvant pyrotinib and letrozole plus dalpiciclib for triple-positive breast cancer
Source: Nat Commun. 2022 Nov 17;13:7043. doi: 10.1038/s41467-022-34838-w (PMC9672048; doi:10.1038/s41467-022-34838-w)
Supplement: Supplementary file 1 — Supplementary Information [file 41467_2022_34838_MOESM1_ESM.pdf]

Supplementary Information

Supplementary Note 1:
Clinical Study Protocol

An open-label, multicentre phase II clinical study of pyrotinib maleate combined with CDK4/6 inhibitor and letrozole in neoadjuvant treatment of stage II-III triple-positive breast cancer (TPBC)

Study Code TPBC-NEO-IIS-SHR6390-PYR

Version NO. 1.5

Version date 23 Jun. 2022

Leading Site Shengjing Hospital of China Medical University

Principal investigator Caigang Liu

Study Coordinating Site

Information	
**Site Name**	Shengjing Hospital Affiliated to China Medical University
**Site Address**	39 Huaxiang Road, Tiexi District, Shenyang 110021
**Principal investigator**	Caigang Liu

Signature page

Signature of the researcher

I will conscientiously perform the duties as a researcher in accordance with the Chinese GCP regulations, and participate in or guide this clinical study. I hereby confirm that I have read this scheme (version number: 1.5; version date: June 23, 2022). I agree to perform relevant duties in accordance with the Chinese law, the Declaration of Helsinki, and the China GCP. This research proposal would not be implemented without the consent of the Ethics Committee, unless measures should be taken for the safety, rights and interests of the subjects.

Research unit: **Shengjing Hospital Affiliated to China Medical University**

Liu Caigang June 23. 2022

______________________ ______________________ ______________________

Principal investigator (Print Body) **Signature Date (year/month/day)**

Catalogue

SYNOPSIS 6
List of Abbreviations 13
Research flow chart 16
1. Research Background 20
2. Study objectives and endpoints 22
3. Research design 23
4. Subject selection and withdrawal 24
4.1 Inclusion criteria 24
4.2 Exclusion criteria 25
4.3 Withdrawal Criteria 26
4.4 Termination criteria 26
4.5 Definition of study completion 26
5 Study drug 26
5.1 Study drug name and source 26
5.2 Method of administration 27
5.3 Management of common adverse events 27
5.4 Dosage adjustment regimen 28
5.5 Drug compliance 30
6 Combination therapy 31
6.1 Drugs prohibited during the study 31
6.2 Medications to be used with caution during the study 31
6.3 Concomitant medications and treatments during the study 31
7 Study procedures 31
7.1 Screening period 32
7.2 Treatment period 33
7.3 Patient perioperative period 34
7.4 End of study treatment/withdrawal from study 34
8 Effectiveness evaluation 35
8.1 Imaging assessment 35
8.2 Primary endpoint 36
8.3 Secondary endpoints 36
9 Safety evaluation 36

9.1 Physical examination and vital signs 37
9.2 laboratory test 37
9.3 ECG 38
9.4 Echocardiography 38
9.5 Adverse event (AE） 38
9.6 Serious adverse event（SAE） 40
9.7 Pregnancy 44
10 Research management 44
10.1 Ethics and Informed Consent 44
10.2 Amendment of the plan 45
10.3 Data management 45
10.4 Plan violation 46
11 Data Analysis and Statistical Methods 46
11.1 Sample size calculation 46
11.2 Statistical Analysis Program 46
12 Case dropout 48
Appendix I: Clinical Staging Criteria for Breast Cancer (Eighth Edition AJCC Breast Cancer TNM Staging） ... 49
Appendix II：Karnofsky Performance Status Scale（KPS） 50
Appendix III：2018 ASCO/CAP Breast Cancer HER2 Testing Guidelines 51
Appendix IV：New response evaluation criteria in solid tumors: Revised RECIST guideline 53
Appendix V：European Organization for Research and Treatment of Cancer Quality of Life Questionnaire – Core 30 ... 58
Appendix VI：European Organization for Research and Treatment of Cancer Quality of Life Questionnaire – Breast Cancer 23 ... 60
Appendix VII：Scoring method of the QLQ-BR23 and EORTC QLQ-C30(raw score and standard score) ... 62

SYNOPSIS

**Study Title**	An open-label, multicenter phase II clinical study of pyrotinib maleate combined with CDK4/6 inhibitor and letrozole in neoadjuvant treatment of stage II-III triple-positive breast cancer (TPBC)
**Leading Site**	Shengjing Hospital of China Medical University
**Principal investigator**	Caigang Liu
**Version date**	23 Jun. 2022
**Version No.**	1.5
**Study Drugs**	Pyrotinib maleate tablets (hereinafter referred to as "pyrotinib")<br>SHR6390（hereinafter referred to as "SHR6390"）<br>Letrozole tablets（hereinafter referred to as "letrozole"）<br>Goserelin（hereinafter referred to as "Goserelin"）
**Background**	Breast cancer is the most prevalent malignancy among women, accounting for 24.2% of all malignancies worldwide, 2.09 million people were diagnosed with breast cancer in 2018, and about 630,000 people died of breast cancer. The prevalence of breast cancer in China accounts for 17.1% of all malignant tumors in women. In 2015, the incidence was about 304,000, and the death rate was about 70,000. Triple-positive breast cancers (TPBCs) refer to estrogen receptor (ER) and progesterone receptor (PR) positive, HER2-overexpressing breast cancers, accounting for 10% to 15% of breast cancers. The NOAH study confirmed that trastuzumab combined with chemotherapy can significantly improve pCR compared with chemotherapy alone. The NeoSphere study confirmed that the pCR rate of trastuzumab and pertuzumab combined with chemotherapy was 45.8%, which was significantly higher than that of trastuzumab combined with chemotherapy (29.0%). pCR rate of HER2 targeted treatment combined with chemotherapy was 20%; pCR rate of trastuzumab/pertuzumab combined with chemotherapy was 26%, which is significantly lower than that in HR-/HER2+ subgroup (63.2%); and the pCR rate of trastuzumab/pertuzumab alone was even lower (6%).<br>The main reasons for drug resistance in triple-positive breast cancer are: ① HER2 dimers with other members of the HER gene family; ② Cross-activation of HER2-related signaling pathways (such as HER1-induced phosphorylation of the PI3K/AKT/mTOR pathway); ③There is a cross-talk between ER-mediated signaling pathways and HER2-mediated signaling pathways. The TBCRC006 study (single-arm, phase II clinical study) explored the application of lapatinib combined with trastuzumab and letrozole in the neoadjuvant treatment of triple-positive breast cancer

	with a pCR rate of 21%, compared with trastuzumab/pertuzumab. The pCR rate of monoclonal antibody and dual-target combined chemotherapy is similar. The TBCRC006 study opened a new idea of neoadjuvant targeted combined endocrine therapy for triple-positive breast cancer, but the pCR rate and residual tumor burden index still need to be further improved.<br>Neoadjuvant endocrine therapy was initially used in elderly breast cancer patients who were not candidates for surgery and chemotherapy. With the advent of aromatase inhibitor (AI) drugs, the efficacy of neoadjuvant endocrine therapy has been further improved, but some patients are still insensitive to endocrine therapy, which may be caused by abnormal expression of cell cycle-related proteases. The cyclin-dependent kinases (CDKs) are a group of key enzymes involved in the cell cycle. CDK4/6 is highly expressed in tumor cells, and inhibition of CDK4/6 can arrest the cell cycle in G1/S phase, thereby inhibiting cell proliferation. The emergence of CDK4/6 inhibitors has brought vitality and hope to endocrine-resistant breast cancer patients (about 30%-40%). The NA-PHER2 study explores neoadjuvant endocrine therapy with trastuzumab/pertuzumab combined with CDK4/6 inhibitor combined with fulvestrant in TPBC, and the pCR rate is 27%.<br>Pyrotinib is an oral, irreversible tyrosine kinase inhibitor (TKI) with simultaneous anti-EGFR/HER1, HER2 and HER4 activity. As a small molecule TKI, pyrotinib can enter the cell membrane, bind to ATP binding sites on cell signaling pathways, and inhibit signaling in downstream cell signaling pathways. Pyrotinib combined with capecitabine is safe and effective in the treatment of HER2-positive advanced breast cancer, with a PFS of up to 18.1 months and an objective response rate of 78.5%. Studies suggest that pyrotinib combined with CDK4/6 inhibitors can significantly inhibit the proliferation of HER2-positive breast cancer cell lines and reduce the activation of pAKT and pHER3 inhibits cell arrest in G0-G1 phase and increases apoptosis. In the mouse animal model, the antitumor activity of pyrotinib combined with CDK4/6 inhibitor was higher than the antitumor activity of either drug alone, and the combined application of the two did not increase the toxicity, which provide a good preclinical model the combination therapy.<br>In conclusion, we envisage that pyrotinib combined with CDK4/6 inhibitor and aromatase inhibitor could serve as a better strategy for neoadjuvant therapy for patients with stage II-III triple-positive breast cancer.
**Study Objectives**	To explore the efficacy and safety of pyrotinib maleate combined with CDK4/6 inhibitor and letrozole in neoadjuvant treatment of stage II-III triple-positive breast cancer (TPBC).
**Study Endpoints**	**Primary endpoint：**

	Pathological complete response rate（tpCR：$ypT_{0\text{-}is}/ypN_0$）<br>**Secondary endpoints**<br>**Efficacy Endpoints**<br>Best overall response rate（BORR）；<br>bpCR：$ypT_{0\text{-}is}$；<br>Residual tumor burden（RCB）；<br>Molecular target：Before treatment and at the time of surgery (required); at 2 weeks of treatment (intentional), at the first efficacy evaluation (after the end of 2 cycles of treatment) at PD/SD (intentional); at the second efficacy evaluation (after the end of 4 cycles of treatment) PD/SD (intentionality)<br>5 years OS，DFS<br>**Safety Endpoints：**<br>Adverse events (AEs) and serious adverse events (SAEs) were based on NCI-CTC AE 5.0 criteria.<br>**Exploratory Endpoints：**<br>Patient reported outcome (PRO) were based on the European Organization for Research and Treatment of Cancer (EORTC) Quality of Life Questionnaire - Core 30 (QLQ-C3 C30; version 3)) questionnaire and Quality of Life Questionnaire - Breast Cancer (QLQ-BR23) module. The changes in scores for different HRQoL dimensions from baseline to every timepoints (at the end of cycle 2 and cycle 4, and before the surgery) were the exploratory endpoints.
**Overall Study Design**	This study adopts a multicenter, single-arm, open-label design, and plans to enroll 89 patients with stage II-III triple-positive breast cancer (TPBC) (Simon two-stage, 67 patients in the first stage), and receive pyrotinib combined with CDK4/6 inhibitor and letrozole as neoadjuvant therapy. The main research purpose is to observe the efficacy and safety of pyrotinib combined with CDK4/6 inhibitor and letrozole neoadjuvant therapy for stage II-III TPBC.<br>After the subjects signed the informed consent, they entered the trial period and received neoadjuvant therapy with pyrotinib combined with CDK4/6 inhibitor and letrozole. Imaging examinations such as breast MRI were reviewed every 2 cycles to evaluate the curative effect. If the curative effect is clear (CR/PR), surgery should be performed within 4 weeks (but at least 2 weeks) after the end of the 5th cycle of neoadjuvant therapy. If the disease is stable (SD) at the first evaluation (after 2 cycles of treatment), it is recommended to continue the treatment with next 2 cycles or to receive an intentional biopsy to guide treatment, but the subjects should be fully informed of the possible risks (poor efficacy/disease). If the disease progresses (PD), subjects will be recommended to withdraw from the study, and receive

treatment recommended by NCCN guidelines. If the second evaluation result (after 4 cycles of treatment) is PD/SD, for operable subjects, surgery or the NCCN guideline-recommended treatment will be recommended; for inoperable subjects, the study will be withdrawn and replaced by the NCCN guideline-recommended protocol. For PD/SD subjects, having received 4 cycles of treatment, an intentional biopsy is recommended to guide subsequent treatment. If the toxicity is not tolerated, the informed consent must be withdrawn or the drug must be discontinued at the discretion of the investigator. The imaging evaluation was performed according to the RECIST1.1 standard, and the evaluation result of the research center was the final result. Within 4 weeks after surgical treatment, the resected tumor tissue and lymph nodes will be examined for histopathology (including the pathology of the tumor margin); the pathological sections will be loaned to the main research unit for unified review.

Follow-up adjuvant therapy:

Targeted therapy: pyrotinib or trastuzumab ± pertuzumab or T-DM1, depending on the disease and patient's intention.

Endocrine therapy: standard endocrine therapy is formulated according to the condition.

Adjuvant chemoradiotherapy: whether it is necessary or not depends on the condition.

Efficacy follow-up: All subjects need to be followed up until tumor progression or death or withdrawal of informed consent, whichever comes first.

Safety follow-up: Follow-up is required until subjects start receiving other anti-tumor drugs; all AEs return to grade 0-1 or baseline level or death, whichever comes first.

Survival follow-up: All subjects underwent survival follow-up until death or withdrawal of informed consent or the end of the trial, whichever occurred first.

The overall design of this study is as follows:

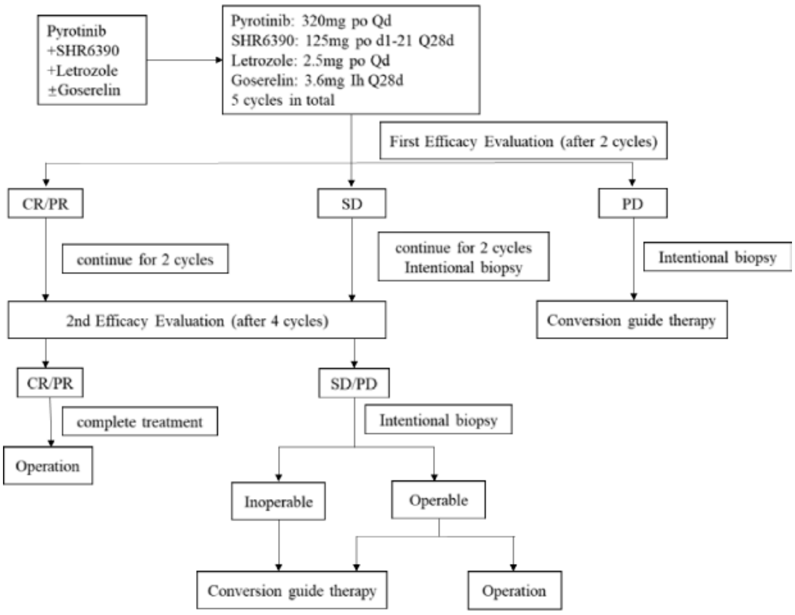

<table>
<tr>
<td>Inclusion Criteria</td>
<td>
Subjects must meet all the following inclusion criteria to be enrolled in this trial:

1. Female patients aged between 18 and 80 years, meet one of the following：
(1) Bilateral oophorectomy in the past, or age ≥ 60 years;
(2) Age < 60 years, natural postmenopausal state (defined as the spontaneous cessation of regular menstruation for at least 12 consecutive months, and no other pathological or physiological cause), E2 and FSH at postmenopausal levels;
(3) Premenopausal or perimenopausal female patients must be willing to receive LHRH agonist therapy during the study period;
2. All patients confirmed by histopathology can be enrolled: Estrogen receptor (ER) positive (>10%), progesterone receptor (PR) positive (>1%), HER2 receptor positive. Following the 2018 version of the ASCO-CAP guidelines for HER2-positive interpretation, HER2 positive was defined as immunohistochemistry (IHC) score of 3+ confirmed by pathology laboratory, or 2+ and positive in situ hybridization (ISH) test (ISH amplification rate ≥2.0);
3. Treatment-naive patients with stage II-III tumors that meet the AJCC 8th edition criteria;
4. KPS score ≥70 points;
5. The functional level of the organ must meet the following requirements:
(1) Bone marrow function
□ ANC ≥ 1.5×109/L (no growth factor used within 14 days);
□ PLT ≥ 100×109/L (without corrective treatment within 7 days);
□ Hb ≥ 100 g/L (without corrective therapy within 7 days);
(2) Liver and kidney function
□ TBIL≤ULN;
□ ALT and AST≤3×ULN;
□ BUN and Cr≤1.5×ULN and creatinine clearance ≥50 mL/min (Cockcroft-Gault formula);
(3) Echocardiography
LVEF ≥ 50%;
(4) 12-lead ECG
QT interval ≤ 480 ms;
6. Able to accept needle biopsy;
7. Voluntarily join the study, sign informed consent, have good compliance and be willing to cooperate with follow-up.
</td>
</tr>
</table>

**Exclusion Criteria**	Anyone who has one of the following conditions cannot participate in the clinical study:<br>1. Received any form of anti-tumor therapy (chemotherapy, radiotherapy, molecular targeted therapy, endocrine therapy, etc.) in the past;<br>2. Receiving any other anti-tumor therapy at the same time;<br>3. Bilateral breast cancer, inflammatory breast cancer or occult breast cancer;<br>4. Stage IV breast cancer;<br>5. Breast cancer without histopathological diagnosis;<br>6. Other malignant tumors have occurred in the past 5 years, except for the cured cervical carcinoma in situ;<br>7. Those with severe insufficiency of vital organs such as heart, liver and kidney;<br>8. Inability to swallow, chronic diarrhea and intestinal obstruction, and other conditions that affect drug taking and absorption;<br>9. Participated in clinical trials of other drugs within 4 weeks before enrollment;<br>10. Known history of allergy to the drug components of this regimen; history of immunodeficiency, including positive HIV test, HCV, active hepatitis B or other acquired, congenital immunodeficiency diseases, or organ transplantation history;<br>11. Ever suffered from any heart disease, including: (1) arrhythmia requiring drug treatment or clinical significance; (2) myocardial infarction; (3) heart failure; (4) any other heart diseases judged by the investigator to be unfit to participate in this trial, etc.;<br>12. Pregnant, lactating female patients, female patients with fertility and positive baseline pregnancy test tests, or female patients of childbearing age who are unwilling to take effective contraceptive measures throughout the trial;<br>13. According to the judgment of the investigator, there are concomitant diseases that seriously endanger the patient's safety or affect the completion of the study (including but not limited to severe hypertension that cannot be controlled by drugs, severe diabetes, active infection, etc.);<br>14. Previous history of a clear neurological or psychiatric disorder, including epilepsy or dementia. Any other conditions for which the patient was deemed unsuitable for participation in this study by the investigator.
**Study Procedures**	From the start of the study, patients with stage II-III triple-positive breast cancer who met the trial inclusion criteria were enrolled. Collect clinical data of patients, such as basic information, tumor-related imaging information, and pathological information (Ki67 and other indicators). Clinicians at each center were responsible for signing the informed consent.<br>Neoadjuvant therapy: pyrotinib combined with SHR6390 and letrozole (goserelin in premenopausal patients) for 20 weeks, every 28 days as a cycle.

	Pyrotinib + SHR6390 + Letrozole +/- Goserelin<br>● Pyrotinib: 320mg, orally, once daily (continuously)<br>● SHR6390: 125mg, orally, once a daily, d1-21, every 28 days as a cycle<br>● Letrozole: 2.5mg, orally, once a daily (continuously)<br>● Goserelin: 3.6mg, subcutaneous injection, every 28 days as a cycle<br>Imaging tests such as breast MRI were reviewed at the end of cycle 2, at the end of cycle 4, and before surgery to assess efficacy.<br>If the response is CR/PR, surgery should be performed within 4 weeks (> 2 weeks) after the end of the 5th cycle of neoadjuvant therapy. If the disease is stable (SD) after the first evaluation (after 2 cycles of treatment), it will be recommended to continue treatment with next 2 cycles or intentional biopsy to guide treatment, but subjects should be fully informed of possible risks (ineffective efficacy/disease progression, etc.); if disease progression (PD), subjects will be recommended to withdraw from the study and accept treatment recommended by NCCN guidelines. If the second evaluation result (after 4 cycles of treatment) is PD/SD, for operable subjects, surgery or the NCCN guideline-recommended protocol will be recommended; for inoperable subjects, the study will be withdrawn, and the NCCN guideline-recommended protocol is recommended. For PD/SD subjects having received 4 cycles of treatment, an intentional biopsy is recommended to guide subsequent treatment.
**Sample Size Determination**	We calculated the sample size based on Simon's admissible two-stage designs. We regarded a proportion of patients with a pCR of 26% or more as proof of efficacy of the combination and of less than 15% as insufficient to continue the assessment. Assuming a risk of α=0·05 (type I error) and β=0·20 (type II error), 61 patients were needed for this first stage. If 12 or more pCR were noted, recruitment proceed for an additional 20 evaluable patients. If 18 or more of 61 patients achieved pCR, the study treatment would be deemed worthy of future study.
**Study Schedule**	Anticipated enrollment of the first subject: July 2020<br>Anticipated enrollment of the last subject: May 2022

List of Abbreviations

Abbreviation	
ADL	activities of daily living
AE	adverse event
AI	aromatase inhibitor
AKP/ALP	alkaline phosphatase
ALB	albumin
ALT	alanine aminotransferase
ANC	absolute neutrophil count
AST	aspartate aminotransferase
AUC	area under concentration time curve
BMI	body mass index
BUN	blood urea nitrogen
CBR	clinical benefit rate
CHOL	cholestenone
CI	confidence interval
CL/F	peak concentration
Cr	creatinine
CR	complete response
CRF	case report form
DBIL	direct bilirubin
DFS	disease free survival
dL	deciliter
DLT	dose limited toxicity
DoR	duration of response
DPD	dihydropyrimidine dehydrogenase
EC	ethics committee
ECOG	Eastern Cooperative Oncology Group
EDC	electronic data collection
EGF	epidermal growth factor
EGFR	epidermal growth factor receptor
ER	estrogen receptor
EORTC	European Organization for Research and Treatment of Cancer
FAS	full analysis set
FISH	fluorescence in situ hybridization
g	gram
GCP	good clinical practice
h	hour
Hb	hemoglobin)
HBV	Hepatitis B Virus
HCV	Hepatitis C Virus
HER2	human epidermal growth factor receptor 2

HIV	Human Immunodeficiency Virus
HR	heart rate
HR	hormone receptor
IBIL	indirect bilirubin
IC_{50}	50% Inhibition Concentration
ITT	intend to treat
IU	international unit
IV	intravenous
kg	kilogram
KPS	Karnofsky Performance Status
L	Liter
LC	lymphocyte count
LDH	lactate dehydrogenase
LVEF	left ventricular ejection fraction
m	meter
min	minute
mg	milligram
ml	milliliter
mm	millimeter
ms	millisecond
MTD	maximum tolerated dose
NCCN	National Comprehensive Cancer Network
NCI-CTC	National Cancer Institute-common terminology criteria
NE	Not evaluable
NMPA	National Medical Products Administration
ORR	objective response rate
OS	overall survival
pCR	pathologic complete response
PD	Pharmacodynamics
PFS	progression free survival
PK	Pharmacokinetics
PLT	platelet
PPS	per protocol set
PR	partial response
PR	progesterone receptor
PRO	Patient reported outcome
QLQ-BR23	Quality of Life Questionnaire - Breast Cancer
QLQ-C30	Quality of Life Questionnaire - Core 30
RBC	red blood cell
RCB	residual cancer burden
RTK	receptor tyrosine kinase
SAE	serious adverse event
SAP	statistical analysis plan

SBP	systolic blood pressure
SD	stable disease
SS	safety set
TBIL	total bilirubin
TG	triglyceride
T_{max}	peak time
TP	plasma total protein
TTP	time to progression
μmol	micromole
ULN	upper normal limit
VEGF	vascular endothelial growth factor
WBC	white blood cell

Research flow chart

	Screening period	Neoadjuvant therapy							Surgery	Study end/exit	Safety follow-up[25]
		C1			C2	C3	C4	C5			
	D-28~D-1	D7±3	D14±3	D28±3	D28±3	D28±3	D28±3	D28±3	Within 4 weeks after C5 end （>2 weeks）		
Sign the informed consent	×										
demographics[1]	×										
Medical history inquiry[2]	×										
Combination therapy[3]	×	×									
Physical examination[4]	×								×（before surgery）	×	
Vital sign[5]	×			×	×	×	×	×	×（before surgery）	×	
KPS score	×		×	×	×	×	×	×	×（before surgery）	×	
Adverse events[6]	From signing informed consent to 28 days after last dose										
Blood routine[7]	×	×	×	×	×	×	×	×	×（before surgery）	×	
Blood chemistry test[8]	×		×	×	×	×	×	×	×（before surgery）	×	
Peripheral blood sample collection[9]	×		×	×	×	×	×	×	×（before surgery）		
Coagulation function test[10]	×	Examination as clinically necessary							×（before surgery）		
Urine routine[11]	×	Examination as clinically necessary							×（before surgery）	×	
Stool routine[12]	×	Examination as clinically necessary							×（before surgery）	×	
Infectious disease screening[13]	×										
Pregnancy test[14]	×								×（before surgery）	×	
12-Lead ECG[15]	×			×	×	×	×	×		×	
echocardiopraphy[16]	×	Examination as clinically necessary									
Primary tumor pathological examination	×										
Molecular target detection[17]	×		×		×		×		×（after surgery）		

	Screening period	Neoadjuvant therapy							Surgery	Study end/exit	Safety follow-up[25]
	D-28~D-1	C1			C2	C3	C4	C5	Within 4 weeks（>2 weeks） after C5 end		
		D7±3	D14±3	D28±3	D28±3	D28±3	D28±3	D28±3			
RCB score									×（after surgery）		
Pathological response rate assessment									×（after surgery）		
Imaging evaluation [18]	×				×		×		×（before surgery）		
Patient reported outcome[19]	×				×		×		×（before surgery）		
Progress/death		×									
Pyrotinib [20]		320mg po qd									
SHR6390[21]		125mg po qd，d1-d21，q4w									
Letrozole [22]		2.5mg po qd									
Goserelin [23]		3.6mg Ih，q4w									
Drug dispensing/recalls [24]		×								×	

Remark：

This study does not allow repeated screening of subjects who have failed previous screening (that is, re-screening is not allowed to sign the informed consent form again; except for subjects who fail screening due to age).

The following inspection items should be completed within the time window (±3 days) listed in the test procedure. If there is an over-window, the reason for the over-window should be recorded in the eCRF. Investigators can increase the inspection items or increase the frequency of video visits according to the clinical conditions of the subjects. Except for the pathological examination, the results of laboratory and auxiliary examinations involved in this study can be examined by outside hospitals, and whether or not to accept the examination results is at the discretion of the researchers.

* Screening period: physical examination, vital signs and laboratory tests (including blood routine, urine routine, liver and kidney function, blood lipids, blood electrolytes, fasting blood glucose, blood HCG, etc.) are conducted within 14 days before the start of study treatment; tumor imaging examination (Breast, chest and abdomen with contrast-enhanced CT or contrast-enhanced MRI, bone scan), 12-lead ECG, echocardiography within 28 days prior to initiation of study treatment. For laboratory test results obtained within 14 days before signing the informed consent form, imaging test results (bone scan allows examination results 2 months before

signing the informed consent form), 12-lead ECG, and cardiac color Doppler ultrasound, if sufficient to support the investigator's judgment of the recipient. If the test subjects meet the inclusion criteria, there is no need to repeat such tests during the screening period, otherwise the test needs to be repeated during the screening period.

1. Demographic data: including initials, gender, ethnicity, marital status, date of birth, height, weight.
2. Medical history inquiry: including past breast cancer medical history and treatment history (clinical/pathological diagnosis, diagnosis time, clinical/pathological stage, HER2/ER/PgR/expression; whether surgery, whether neoadjuvant therapy, whether adjuvant therapy, whether radiotherapy), history of tumors other than breast cancer, history of smoking and drinking (whether smoking or drinking, whether you have quit), history of drug allergy (name of drug, allergic symptoms), previous disease or concomitant disease/symptom (name of disease/symptom, duration, whether or not treatment and outcomes), bowel habits (frequency).
3. Concomitant medication: record the concomitant medication from the screening period to the end of the study treatment. The combined medication record should include the name of the drug, the dose of the drug, the route of administration, the frequency of administration, the purpose of administration, and the start and end dates. If the subject starts new systemic anti-tumor therapy during the safety follow-up period, only the concomitant/concomitant therapy for adverse events related to the study drug will be recorded.
4. Physical examination: including evaluation of head, lymph nodes, eyes, ears, nose, throat, skin, oral cavity, musculoskeletal system, respiratory system, cardiovascular system, abdomen, genitourinary system, nervous system and mental state. Screening period, before surgery and at study end/withdrawal (If not done within the previous 7 days) 1 inspection.
5. Vital signs: including respiratory rate, pulse, heart rate, blood pressure and body temperature. 1 examination at screening, on the last day of each cycle during neoadjuvant therapy, before surgery, and at study end/withdrawal if not performed within the previous 7 days.
6. Adverse events: From the time the patient signed the consent form until at least 28 days after the last medication, the adverse events, concomitant medications/treatments and unscheduled examinations during the period were recorded in detail, until the adverse events were relieved or reached a stable level deemed unrelievable by the investigator, or the subjects started New antitumor therapy or lost to follow-up.
7. Blood routine examination: including hemoglobin, red blood cell count, platelet count, white blood cell count, neutrophil count and lymphocyte count. At Screening, Days 7, 14, 28 of the first cycle of neoadjuvant therapy, and on the last day of each subsequent cycle, before surgery, and at the end/withdrawal of the study (if not done within the previous 7 days). If necessary, the investigator can increase the frequency of examinations according to the clinical conditions of the subjects.
8. Blood biochemical tests: including sodium, potassium, chloride, calcium, phosphorus, magnesium, cholesterol, triglycerides, glucose, BUN or urea, creatinine, uric acid, total protein, albumin, alkaline phosphatase, ALT, AST, GGT, total bilirubin and direct bilirubin, etc. Blood chemistry should be performed during the screening period, on days 14 and 28 of the first cycle of neoadjuvant therapy, on the last day of each subsequent cycle, before surgery, and at the end/withdrawal of the study (if not done within the previous 7 days). If necessary, the investigator can increase the frequency of examinations according to the clinical conditions of the subjects
9. Peripheral blood sample collection: 8 mL of anticoagulant was collected during the screening period, at 2 weeks of treatment, at the end of each cycle, and before surgery.
10. Coagulation function test: including INR, APTT, PT, FIB, TT. The screening period and the operation were performed once each. During the study period, the investigator decides to check according to clinical practice or needs.
11. Urine routine: including urine protein, urine sugar, urine red blood cells, and urine white blood cells. 1 examination each at screening, before surgery, and at end/withdrawal of the study (if not performed within the previous 7 days). During the study period, the investigator decides to check according to clinical practice or needs. If urine routine shows urine protein ++ or above, please check 24h urine protein

quantification.

12. Stool routine: including fecal occult blood. 1 at screening, before surgery, and at end/withdrawal of treatment (if not done within the previous 7 days). During the study period, the investigator decides to check according to clinical practice or needs.
13. Infectious disease screening: including five hepatitis B, HIV antibody, HCV antibody testing. 1 test during the screening period. If the hepatitis B and C test results are abnormal, the investigator should determine whether to perform the virus replication (HBV-DNA, HCV-RNA) test.
14. Pregnancy test: Female subjects of childbearing age should undergo blood HCG testing to rule out pregnancy during the screening period, before surgery, and at the end/withdrawal of treatment (if not performed within the previous 7 days).
15. 12-lead ECG: 1 each at screening, on the last day of each cycle of neoadjuvant therapy, before surgery, and at the end/withdrawal of the study (if not performed within the previous 7 days). If the QTc interval increases by >30 msec from the baseline, or if the absolute value of the QTc interval is ≥480 msec in any of the specified ECG measurements, 2 additional ECG examinations (at least 10 minutes apart) are required.
16. Echocardiography: once during the screening period. During the study period, if symptoms such as chest pain, palpitations and abnormal electrocardiograms occur, additional tests can be taken as appropriate.
17. Tumor histopathological sampling: screening period (required), at 2 weeks of treatment (intentional), at the first efficacy evaluation (after the end of 2 cycles of treatment) at PD/SD (intentional), at the second efficacy evaluation (Tumor samples were collected by needle biopsy at SD (intentional) at the end of 4 cycles of therapy, and by surgical excision at surgery (required). Each test center is responsible for collecting samples, and the Shengjing Hospital of China Medical University uniformly measures Ki67, [redacted] level.
18. Tumor imaging evaluation: Imaging evaluation will be performed according to the principles of the RECIST version 1.1 criteria. The screening period includes breast, chest, abdomen, and bone scans. According to clinical conditions, imaging examinations of the brain, neck, and pelvis can be added to exclude metastasis. Available with CT or MRI and other methods, breast tumor evaluation chooses MRI. Evaluations should be performed every two cycles during neoadjuvant therapy and before surgery under the same conditions as baseline examinations (contrast media use, etc.) (bone scan at the end of neoadjuvant therapy). Unplanned imaging studies may be performed when disease progression (e.g., worsening symptoms) is suspected.
19. Patient reported outcome (PRO)： Patient reported outcome (PRO) will be assessed by the European Organisation for Research and Treatment of Cancer (EORTC) Quality of Life Questionnaire-Core 30 (QLQ-C30; version 3) questionnaire and Quality of Life Questionnaire - Breast Cancer (QLQ-BR23) module. Assessment should be performed at the baseline, the end of cycle2 and cycle 4, and before surgery. To avoid the influence of imaging examination results on the PRO assessment, the PROs should be assessed before imaging examination.
20. Pyrotinib: 320mg, orally, once a day.
21. SHR6390: Take orally on an empty stomach, once a day, 125 mg each time, for 3 weeks, stop for 1 week, and 4 weeks as a cycle. For the requirements of taking SHR6390 on an empty stomach, take SHR6390 tablets orally on an empty stomach in the morning with warm water, and fast for 1 hour before and 1 hour after taking the medicine during continuous administration.
22. Letrozole: 2.5mg each time, once a day.
23. Goserelin: 3.6mg, subcutaneous injection, 4 weeks as a cycle.
24. Drug distribution/recovery: Except for the first cycle where only drugs are distributed and only drugs are recovered at the end of treatment, the drugs are recovered and distributed at the end of each cycle, and

corresponding records are made.

25. Safety follow-up: At least 28 days after the last study medication, the investigator can select blood routine, blood biochemistry, electrocardiogram, echocardiography, physical examination, vital signs, KPS score and other clinical evaluation measures according to the specific conditions of the subjects, as Unscheduled inspection records. Drug-related adverse events should be followed up until disappearance, remission to baseline level ≤ grade 1, stable status, or reasonable explanation (eg loss to follow-up, death).

1. Research Background

Breast cancer is the most common malignant tumor in women, accounting for 24.2% of all malignant tumors. In 2018, 2.09 million people were diagnosed with breast cancer worldwide, and about 630,000 people died of breast cancer. The prevalence of breast cancer in my country accounts for 17.1% of all malignant tumors in women. In 2015, the incidence was about 304,000, and the death rate was about 70,000. Triple-positive breast cancers (TPBCs) refer to estrogen receptor (ER) and progesterone receptor (PR) positive, HER2-overexpressing breast cancers, accounting for 10% to 15% of breast cancers. The NOAH study confirmed that trastuzumab combined with chemotherapy can significantly improve pCR compared with chemotherapy alone, establishing the status of trastuzumab as a neoadjuvant targeted therapy. The NeoSphere study confirmed that the pCR rate of trastuzumab and pertuzumab combined with chemotherapy was 45.8%, which was significantly higher than that of trastuzumab combined with chemotherapy (29.0%). Target combined chemotherapy pCR rate was 20%; trastuzumab/pertuzumab dual target combined chemotherapy pCR rate was 26%, significantly lower than the HR-/HER2+ subgroup (63.2%); The pCR rate of dual target therapy alone was even lower (6%). The main reasons for drug resistance in triple-positive breast cancer are: ① HER2 dimers with other members of the HER gene family; ② cross-activation of HER2-related signaling pathways (such as HER1-induced phosphorylation of PI3K/AKT/mTOR pathway); ③ There is a crossover between the ER-mediated signaling pathway and the HER2-mediated signaling pathway. The TBCRC006 study (single-arm, phase II clinical study) explored the application of lapatinib combined with trastuzumab and letrozole in neoadjuvant treatment of triple-positive breast cancer with a pCR rate of 21%, which was comparable to that of trastuzumab/pertuzumab. The pCR rate of monoclonal antibody and dual-target combined chemotherapy is similar. The TBCRC006 study opens up a new idea of neoadjuvant targeted combined endocrine therapy for triple-positive breast cancer, but its pCR rate and residual tumor burden still need to be further improved.

Neoadjuvant endocrine therapy was initially used in elderly breast cancer patients who were not candidates for surgery and chemotherapy. With the advent of aromatase inhibitor (AI) drugs, the efficacy of neoadjuvant endocrine therapy has been further improved, but some patients are still insensitive to endocrine therapy, which may be caused by abnormal expression of cell cycle-related proteases. endocrine resistance. The cyclin-dependent kinases CDKs are a group of key enzymes involved in the cell cycle. CDK4/6 is highly expressed in tumor cells, and inhibition of CDK4/6 can arrest the cell cycle in G1/S phase, thereby inhibiting cell proliferation. The emergence of CDK4/6 inhibitors has brought vitality and hope to endocrine-resistant breast cancer patients (about 30%-40%). The NA-PHER2 study explored the use of trastuzumab/pertuzumab combined with CDK4/6 inhibitor combined with fulvestrant neoadjuvant endocrine therapy for triple-positive breast cancer, and the pCR rate was 27%.

Pyrotinib is an oral, irreversible tyrosine kinase inhibitor (TKI) with simultaneous anti-EGFR/HER1, HER2 and HER4 activity. As a small molecule TKI, pyrotinib can enter the cell membrane, bind to ATP binding sites on cell signaling pathways, and inhibit signaling in downstream cell signaling pathways. Pyrotinib combined with capecitabine is safe and effective in the treatment of HER2-positive advanced breast cancer, with a PFS of up to 18.1 months and an objective response rate of 78.5%. Studies have shown that pyrotinib combined with CDK4/6 inhibitors can significantly inhibit the proliferation of HER2-positive breast cancer cell lines, reduce the activation of pAKT and pHER3, inhibit cell arrest in G0-G1 phase, and increase apoptosis. In the mouse animal model, the antitumor activity of pyrotinib combined with CDK4/6

inhibitor was higher than the antitumor activity of either drug alone, and the combined application of the two did not increase the toxicity. Combination therapy with inhibitors provides a good preclinical model.

SHR6390 is a CDK4/6 kinase inhibitor developed by Jiangsu Hengrui Medicine Co., Ltd. Preclinical data show that SHR6390 selectively inhibits CDK4/6 kinase activity, so that its complex with Cyclin D cannot phosphorylate downstream Rb proteins, preventing cells from entering S phase from G1 phase, thereby inhibiting cell proliferation and anti-tumor effects. In the in vitro enzymatic assay, SHR6390 showed obvious selectivity for CDK4/CDK6 inhibition.

There are currently two ongoing clinical studies of SHR6390 in combination with pyrotinib for the treatment of advanced breast cancer. One of the Phase II clinical studies has enrolled 8 patients, and common adverse reactions are diarrhea, decreased white blood cell count, decreased neutrophil count, anemia, oral mucositis, nausea and vomiting, and increased direct bilirubin. Adverse events > Grade 3 were: decreased neutrophil count, decreased white blood cell count, diarrhea, increased direct bilirubin, nausea, vomiting, and thrombocytopenia. Only 1 case of SAE occurred and may not be related to the study drug. Compared with SHR6390 monotherapy, the combination of pyrotinib did not significantly increase the incidence of neutropenia. SHR6390 in combination with pyrotinib also did not significantly increase the incidence of severe diarrhea compared with pyrotinib monotherapy. Overall, the combination of pyrotinib with SHR6390 has an acceptable safety profile for advanced breast cancer.

Taken together, we envision that pyrotinib combined with SHR6390 combined with aromatase inhibitors may provide a better strategy for neoadjuvant therapy in patients with stage II-III triple-positive breast cancer. Since there is no precedent for pyrotinib combined with SHR6390 combined with aromatase inhibitor in the neoadjuvant treatment of triple-positive breast cancer patients, the exploratory single-arm design is more reasonable, and the efficacy and safety of this trial scheme will be initially explored before the trial. Expand the scale of the study and design a randomized controlled study.

2. Study objectives and endpoints

Research purposes	Study endpoint
The main purpose	Primary endpoint
To explore the efficacy and safety of pyrotinib maleate combined with CDK4/6 inhibitor and letrozole in neoadjuvant treatment of stage II-III triple-positive breast cancer (TPBC).	Pathological complete response rate（tpCR: ypT_{0-is}/ypN_0）
	Secondary endpoints
	Effectiveness Metrics:<br>Best overall response rate（BORR）;<br>bpCR: ypT_{0-is} ;<br>Residual tumor burden（RCB）;<br>Molecular target: Before treatment and at the time of surgery (required); at 2 weeks of treatment (intentional), at the first efficacy evaluation (after the end of 2 cycles of treatment) at PD/SD (intentional); at the second efficacy evaluation (after the end of 4 cycles of treatment) PD/SD (intentionality)<br>Ki67;<br>[redacted]<br>5 years OS, DFS<br>**Safety indicators:**<br>Adverse events (AEs) and serious adverse events (SAEs) were based on NCI-CTC AE 5.0 criteria.<br>**Exploratory research:**<br>Collect and save 8ml of anticoagulant at baseline, at 2 weeks of treatment, at the end of each cycle, and at surgery for exploratory studies of factors that may influence or predict efficacy.<br>Patient reported outcome (PRO) were based on the European Organisation for Research and Treatment of Cancer (EORTC) Quality of Life Questionnaire - Core 30 (QLQ-C30; version 3) questionnaire and Quality of Life Questionnaire - Breast Cancer (QLQ-BR23) module.The

	changes in scores for different HRQoL dimensions from baseline to every timepoints(at the end of cycle 2 and cycle 4, and before the surgery) were the exploratory endpoints.

3. Research design

This study adopts a multicenter, single-arm, open-label design, and intends to enroll 89 patients with stage II-III triple-positive breast cancer (TPBC) (Simon two-stage, 67 patients in the first stage), receiving pyrotinib combined with CDK4/ 6 inhibitor and letrozole neoadjuvant therapy. The main research purpose is to observe the efficacy and safety of pyrotinib combined with CDK4/6 inhibitor and letrozole neoadjuvant therapy for stage II-III triple-positive breast cancer (TPBC).

After the subjects signed the informed consent, they entered the trial period and received neoadjuvant therapy with pyrotinib combined with CDK4/6 inhibitor and letrozole. Imaging examinations such as breast MRI were reviewed every 2 cycles to evaluate the curative effect. If the response is clear (CR/PR), surgery should be performed within 4 weeks (more than 2 weeks) after the end of the 5th cycle of neoadjuvant therapy. If the first evaluation (after 2 cycles of treatment) has stable disease (SD), it is recommended to continue treatment with next 2 cycles or to receive an intentional biopsy to guide treatment, but the subjects should be fully informed of the possible risks (poor efficacy/disease); If the disease progresses (PD), withdraw from the study and receive treatment recommended by NCCN guidelines. If the second evaluation (after 4 cycles of treatment) is PD/SD, for operable subjects, surgery is recommended or the NCCN guideline-recommended protocol is replaced; for inoperable subjects, the study is withdrawn and the NCCN guideline-recommended protocol is replaced. For subjects receiving 4 cycles of treatment for PD/SD, an intentional biopsy is recommended to guide subsequent therapy. If the toxicity is not tolerated, the informed consent must be withdrawn or the drug must be discontinued at the discretion of the investigator. The imaging evaluation was performed according to the RECIST1.1 standard, and the evaluation result of the research center was the final result. Within 4 weeks after surgical treatment, the resected tumor tissue and lymph nodes will be examined for histopathology (including the pathology of the tumor margin); the pathological sections will be loaned to the main research unit for unified review.

Follow-up adjuvant therapy：

Targeted therapy: pyrotinib or trastuzumab ± pertuzumab or T-DM1, depending on the disease and patient's intention.

Endocrine therapy: standard endocrine therapy is formulated according to the condition.

Adjuvant chemoradiotherapy: whether it is necessary or not depends on the condition.

Efficacy follow-up: All subjects need to be followed up until tumor progression or death or withdrawal of informed consent, whichever comes first.

Safety follow-up: Follow-up is required until subjects start receiving other anti-tumor drugs; all AEs return to grade 0-1 or baseline level or death, whichever comes first.

Survival follow-up: All subjects underwent survival follow-up until death or withdrawal of informed consent or the end of the trial, whichever occurred first.

The overall design of this study is as follows:

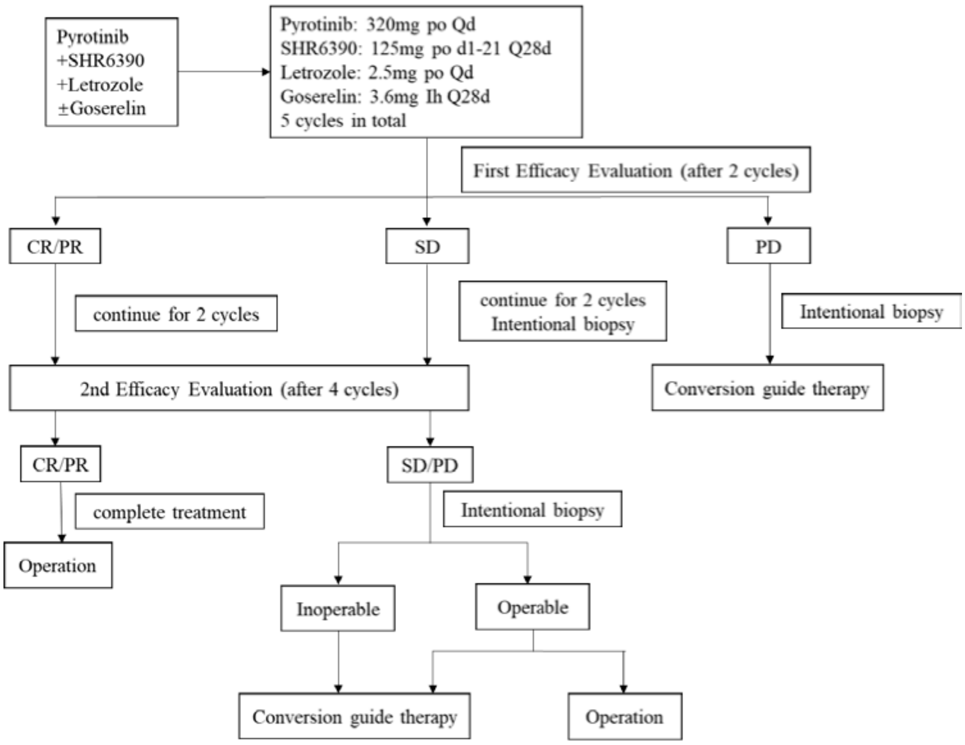

4. Subject selection and withdrawal

4.1 Inclusion criteria

Subjects must meet all of the following inclusion criteria to be enrolled in this trial:

1. Female patients aged ≥ 18 and ≤80 years old, meet one of the following:
   (1) Bilateral oophorectomy in the past, or age ≥ 60 years;
   (2) Age < 60 years, natural postmenopausal state (defined as the spontaneous cessation of regular menstruation for at least 12 consecutive months, and no other pathological or physiological cause), E2 and FSH at postmenopausal levels;
   (3) Premenopausal or perimenopausal female patients must be willing to receive LHRH agonist therapy during the study period;
2. All patients confirmed by histopathology can be enrolled: Estrogen receptor (ER) positive (>10%), progesterone receptor (PR) positive (>1%), HER2 receptor positive. Following the 2018 version of the ASCO-CAP guidelines for HER2-positive interpretation, HER2 positive was defined as immunohistochemistry (IHC) score of 3+ confirmed by pathology laboratory, or 2+ and positive in situ hybridization (ISH) test (ISH amplification rate ≥2.0);
3. Treatment-naive patients with stage II-III tumors that meet the AJCC 8th edition criteria;
4. KPS score ≥70 points;
5. The functional level of the organ must meet the following requirements:
   (1) Bone marrow function

- ✓ ANC ≥ 1.5×109/L (no growth factor used within 14 days);
- ✓ PLT ≥ 100×109/L (without corrective treatment within 7 days);
- ✓ Hb ≥ 100 g/L (without corrective therapy within 7 days);

(2) Liver and kidney function

- ✓ TBIL≤ULN;
- ✓ ALT and AST≤3×ULN;
- ✓ BUN and Cr≤1.5×ULN and creatinine clearance ≥50 mL/min (Cockcroft-Gault formula);

(3) Echocardiography

LVEF ≥ 50%;

(4) 12-lead ECG

QT interval ≤ 480 ms;

6. Able to accept needle biopsy;
7. Voluntarily join the study, sign informed consent, have good compliance and be willing to cooperate with follow-up.

4.2 Exclusion criteria

Anyone who has one of the following conditions cannot participate in the clinical study:

1. Received any form of anti-tumor therapy (chemotherapy, radiotherapy, molecular targeted therapy, endocrine therapy, etc.) in the past;
2. Receiving any other anti-tumor therapy at the same time;
3. Bilateral breast cancer, inflammatory breast cancer or occult breast cancer;
4. Stage IV breast cancer;
5. Breast cancer without histopathological diagnosis;
6. Other malignant tumors have occurred in the past 5 years, except for the cured cervical carcinoma in situ;
7. Those with severe insufficiency of vital organs such as heart, liver and kidney;
8. Inability to swallow, chronic diarrhea and intestinal obstruction, and other conditions that affect drug taking and absorption;
9. Participated in clinical trials of other drugs within 4 weeks before enrollment;
10. Known history of allergy to the drug components of this regimen; history of immunodeficiency, including positive HIV test, HCV, active hepatitis B or other acquired, congenital immunodeficiency diseases, or organ transplantation history;
11. Ever suffered from any heart disease, including: (1) arrhythmia requiring drug treatment or clinical significance; (2) myocardial infarction; (3) heart failure; (4) any other heart diseases judged by the investigator to be unfit to participate in this trial, etc.;
12. Pregnant, lactating female patients, female patients with fertility and positive baseline pregnancy test tests, or female patients of childbearing age who are unwilling to take effective contraceptive measures throughout the trial;

13. According to the judgment of the investigator, there are concomitant diseases that seriously endanger the patient's safety or affect the completion of the study (including but not limited to severe hypertension that cannot be controlled by drugs, severe diabetes, active infection, etc.);

14.Previous history of a clear neurological or psychiatric disorder, including epilepsy or dementia. Any other conditions for which the patient was deemed unsuitable for participation in this study by the investigator.

4.3 Withdrawal Criteria

4.3.1 Criteria for subject withdrawal

1. The subject voluntarily withdraws the informed consent at any time;
2. After randomization, it was found that the subjects seriously violated the inclusion and exclusion criteria;
3. Medical imaging progress or clinical progress;
4. There are any clinical adverse events, abnormal laboratory tests or other medical conditions, which may cause the subjects to continue to use the drug and may no longer benefit;
5. During the course of the research, the subject has a pregnancy event;
6. Serious violation of the trial protocol, and the investigator's assessment believes that the treatment should be terminated;
7. Other reasons considered by the investigator to be unable to continue the study drug treatment.

4.3.2 Handling of withdrawal subjects

Investigators can suggest or provide new or alternative treatments to patients based on their actual conditions.

4.4 Termination criteria

The study termination criteria include but are not limited to the following:

1. Unexpected, significant or unacceptable risks to the subject are found;
2. Major mistakes were found in the plan during the execution of the test;
3. The study drug/trial treatment is ineffective, or it is meaningless to continue the trial;
4. Completion of the trial is extremely difficult due to reasons such as severe delays in subject selection or frequent protocol deviations.

If the principal investigator terminates or suspends the clinical study in advance, the subjects must be informed immediately, and the ethics committee shall be reported in writing with the specific reasons.

4.5 Definition of study completion

End of study was defined as either the last subject completed surgery or the investigator deemed it necessary to end the trial early.

5 Study drug

5.1 Study drug name and source

SHR6390 is an unlisted drug CDK4/6 inhibitor produced by Jiangsu Hengrui Pharmaceutical Co., Ltd., produced and supplied by Jiangsu Hengrui Pharmaceutical Co., Ltd.;

Pyrotinib maleate tablets, letrozole and goserelin are all commercially available drugs.

5.2 Method of administration

Pyrotinib maleate: 320 mg once daily, orally within 30 minutes after breakfast, continuously.

SHR6390: 125 mg once daily, 3 weeks on, 1 week off, 4 weeks as a cycle. It is recommended to take it at about the same time every day

Taking the medicine with warm water, it is recommended to fast on an empty stomach and at least 1 hour before and after taking the medicine. Letrozole: 2.5mg, orally, once a day, continuous administration.

For non-menopausal women, it is necessary to combine with a drug-induced ovarian function inhibitor: goserelin: 3.6 mg, 4 weeks as a cycle, subcutaneous injection. The above medication can be adjusted according to the program according to the adverse reactions of the subjects. Subject continued medication until surgery, withdrawal informed, or

The doctor believes that the medication must be discontinued. The date of the medication cycle is determined from the first day of the subjects taking the medication. During the trial, if any oral medication is suspended, missed, or vomited, they will continue to take the medication according to the schedule, and no supplement or periodic adjustment will be given. However, it should be recorded in the original data in detail: If a drug is missed, the time when the missed drug should be taken and the reason for the missed dose should be recorded in detail; if there is less use due to various reasons such as adverse drug reactions, it should be recorded in the original data. in the medical record.

5.3 Management of common adverse events

In the event of adverse events during the study, the investigators took active symptomatic treatment according to the actual clinical situation and clinical routine procedures, and made detailed records of the combined treatment and medication in the course of disease and CRF. According to the data from the preliminary clinical trial of pyrotinib, the single agent of pyrotinib was well tolerated. The incidence of grade 3/4 adverse events was 11.1% (1/9) in the 320 mg dose group. Dose adjustment of pyrotinib was recommended in the study with reference to Table 1. Investigators should carry out medical treatment according to the actual clinical situation. The following treatment methods are for reference:

Diarrhea: Before subjects start oral test drug, the investigator should inform in detail the possibility of diarrhea and the treatment measures for diarrhea. When diarrhea occurs, symptomatic treatment should be given first, followed by close follow-up or observation (≤14 days). It is clinically recommended to start oral montmorillonite powder on the day of diarrhea, 3g/bag, 3 times/day; for severe diarrhea, oral or intravenous electrolytes can be administered, loperamide can be taken, and one tablet can be taken each time the stool is not formed. (2mg), the maximum dose is 8 capsules (16mg) / day, until the diarrhea stops more than 12 hours. For grade III diarrhea that still cannot be relieved or grade I to II with complications, it is recommended to suspend pyrotinib. Dosing should be resumed after the adverse events have recovered to grade I.

Hand-foot syndrome and rash: give symptomatic treatment first and follow up closely. Suggested symptomatic and supportive care: Intensive skin care, keep skin clean, avoid secondary infection; avoid pressure or friction; use

moisturizer or lubricant, topical lotion or lubricant containing urea and corticosteroids; topical as necessary Antifungal or antibiotic treatment.

Abnormal liver function: The investigator shall give symptomatic treatment or observation of liver protection according to the subjects and AE conditions, and the frequency of blood biochemical examination shall be increased according to clinical needs.

Vomiting: give symptomatic treatment first and follow up closely. If the vomiting is close to the time of taking the medicine on the same day, the occurrence time of the vomiting should be recorded in detail. However, regardless of whether the vomiting affects the absorption of the study drug, the drug will continue to be taken according to the plan cycle after that, without supplementary service or cycle adjustment.

Cardiotoxicity: QT interval prolongation: Before initiating pyrotinib, subjects should be corrected for hypokalemia, hypomagnesemia, or hypocalcemia. When the patient has the following conditions, the medication process of pyrotinib should be vigilant:

1) underlying cardiac diseases or special conditions: such as previous accumulation of high-dose anthracycline therapy;

2) congenital long QT syndrome;

3) low Kalemia, hypocalcemia, hypomagnesemia; 4) Simultaneous use of 2 or more drugs that cause QT interval prolongation.

Decreased left ventricular ejection fraction (LVEF): Before initiating pyrotinib therapy, LVEF should be confirmed to be within the normal range. During the study, LVEF should be regularly monitored to ensure that LVEF does not fall below the lower limit of normal.

Neutropenia: blood routine test is required before subjects start using the test drug, if ANC count <1000/mm3, platelets <50,000/ mm3, the SHR6390 is not recommended. Do not withhold SHR6390 unless grade 3 neutropenia or febrile neutropenia occurs, if grade 3 neutropenia persists for >1 week after discontinuation or repeats grade 3 intermediate on day 1 of subsequent cycles neutropenia, a dose reduction of SHR6390 is recommended when restarting treatment.

5.4 Dosage adjustment regimen

5.4.1 Suspension and dose reduction of pyrotinib

The investigators chose to suspend or discontinue pyrotinib based on the type and duration of clinical adverse events. Multiple suspensions and multiple dose adjustments of pyrotinib were allowed in the trial. The dose of pyrotinib was adjusted according to a gradient of 320 mg, 240 mg, and 160 mg. If the subject still cannot tolerate the dose of 160 mg, the subject should withdraw from the study.

For each suspension, the administration should be resumed after the adverse events having recovered to grade 0-I and the complications having disappeared. In addition, the suspension time should be included in the administration cycle. The cumulative suspension time for a single dose of pyrotinib should not exceed 14 days in order to ensure that the subject receives the drug strength of the treatment. If the suspension of pyrotinib due to adverse events exceeds the above criteria, the subjects should be excluded from the study.

Table 1. Recommended Pyrotinib Dosage Adjustment for Adverse Events

Adverse event	**CTCAE 5.0* Grade**	Treatment of the course of treatment (after active clinical treatment or observation)	Resume pyrotinib dose adjustment after suspension*
Diarrhea	Grade 3 or Grade 1-2 with complications (Grade ≥2 nausea or vomiting, fever, neutropenia, blood in the stool, or dehydration)	Withhold dosing until recovery to Grade≤ 1 and complication resolves	the first time：320 mg<br>the second time：240 mg<br>the third time：160mg
	Grade 4	Permanently disable	——
Left ventricular ejection fraction (LVEF) decline	Clinically significant grade ≥2 LVEF decline/LVEF below the lower limit of normal (including asymptomatic decline in LVEF ≥10% and LVEF <50%, or heart failure)	Permanently disable	——
Abnormal liver function	Grade ≥3 ALT or AST elevation with total bilirubin ≤2×ULN	Withhold pyrotinib until recovery to ≤Grade 1	the first time：320 mg<br>the second time：240 mg<br>the third time：160mg
	Grade ≥2 ALT or AST elevation with total gallbladder Elevated erythrocytes > 2×ULN	Permanently disable	——
Other adverse events	Nonhematologic adverse events of grade ≥2 (excluding hair loss, fatigue, asthenia, etc.) and hematological adverse events of grade ≥3	Withhold pyrotinib until recovery to Grade≤ 1	the first time：320 mg<br>the second time：240 mg<br>the third time：160mg

LVEF, Left Ventricular Ejection Fraction。

* Grades for hand-foot skin reactions and other adverse events not covered can be found in 9.5.2. The investigators give clinical active treatment or observation (≤14 days) according to the conditions of the subjects and adverse events. If it still exists, it is recommended to adjust the drug with reference to this table.

5.4.2 Suspension and dose downregulation of SHR6390

After a toxic reaction occurs, the doctor can judge according to the situation and give corresponding treatment. The specific treatment principles are suggested as follows:

When there is a toxicity clearly related to the study drug, the investigator will deal with it according to clinical

manifestations. After recovery to ≤ grade I (or the investigator judges that the adverse events of ≤ grade II are tolerable to the subjects and have no obvious safety risk), the drug can be re-administered. If the same adverse event recurs again, the investigator will suspend and adjust the dose according to the judgment of the investigator, and the investigator will protect the safety of the subjects to the greatest extent. If the toxicity cannot be recovered within 4 weeks after drug suspension, the subjects should withdraw from the study in principle. The dosing period should be included in the dosing cycle.

Table 2. SHR6390 Dosage Adjustment Program Recommendation Table

Adverse event NCI CTCAE 5.0	Grade			
	I	II	III	IV
hematological toxicity	maintain the original dose	maintain original dose	Suspend the medication, treat symptomatically, and recover to Grade ≤ I. According to the judgment of the investigator, the original dose can be maintained or the dose can be reduced by one dose in this cycle and subsequent cycles.	Suspend medication, treat symptomatically, and recover to grade I or below. According to the investigator's judgment, the dose will be reduced by one dose in this cycle and subsequent cycles
non-hematological toxicity	maintain the original dose	Maintain original dose or suspend medication, treat symptomatically, and recover to grade I or below. According to the investigator's judgment, the original dose can be maintained or reduced in this cycle and subsequent cycles. a dose	Suspend medication, treat symptomatically, and recover to Grade ≤ I. According to the investigator's judgment, the dose will be reduced by one dose in this cycle and subsequent cycles	Permanently discontinue medication, withdraw from study
Febrile neutropenia	-	-	Suspend medication, treat symptomatically, and recover to Grade ≤ I. According to the investigator's judgment, the dose will be reduced by one dose in this cycle and subsequent cycles	Permanently discontinue medication, withdraw from study

During the trial, multiple suspensions and multiple dose adjustments of the test drug are allowed. SHR6390 recommends adjusting the dose according to a gradient of 125 mg, 100 mg, and 75 mg. If there are still intolerable or uncorrectable adverse reactions after adjusting to the lowest dose gradient, the drug should be stopped.

5.5 Drug compliance

Calculation of dose compliance:

% compliance = number of tablets taken / number of tablets expected to be taken ×100.

If the percent compliance calculated by the above formula is less than 80% or greater than 120%, the subject is considered non-compliant with the dosing. If a subject's compliance falls outside the above ranges, it will be recorded as a protocol deviation.

6 Combination therapy

6.1 Drugs prohibited during the study

During treatment, antitumor drugs and adjuvant drugs related to tumor treatment, including antitumor traditional Chinese medicines and immune preparations, should be stopped.

6.2 Medications to be used with caution during the study

If the subjects have adverse reactions, they should be closely observed, and active symptomatic treatment should be given if necessary. The following drugs were used with caution during the study:

- Drugs that interfere with liver P450 enzymes:
  1. CYP3A4 inducers (catamizine, rifampicin and phenobarbital) and inhibitors (ketoconazole, itraconazole, erythromycin and clarithromycin);
  2. Substrates of CYP3A4 (simvastatin, cyclosporine and pimidine);
  3. Other drugs metabolized by CYP3A4 (such as benzodiazepines, dihydropyridine, calcium ion antagonists and HMG-COA reductase inhibitors);
  4. Substrates of CYP2C9 (diclofenac, phenytoin, piroxicam, S-warfarin, and tolbutamide) and substrates of CYP2C19 (diazepam, imipramine, lansoprazole, and S-mephentoin);
- Drugs that prolong the QT interval:
  Drugs including antibiotics, antiarrhythmics, antipsychotics, antifungals, antimalarial drugs, and antidepressants (eg, clarithromycin, quinidine, risperidone, fluconazole, mefloquine, amitripty Lin, Azithromycin, Sotalol, Fluphenazine, Ketoconazole, Chloroquine, Imipramine, Erythromycin, Amiodarone, Droperidol, Clomipramine, Roxithromycin, Disopyramide, Haloperidol, dotipine, metronidazole, procainamide, thioridazine, doxepin, moxifloxacin, pimozide, olanzapine, and clozapine).
- Use products containing St. John's wort (Hypericum perforatum)

6.3 Concomitant medications and treatments during the study

The patients can receive the best supportive treatment, and the clinical co-morbidities and various AEs should be actively treated. All drugs used in combination should be recorded in the CRF in strict accordance with the provisions of GCP. Concomitant medications/treatments should be recorded from 4 weeks prior to study treatment until the end of the safety follow-up.

7 Study procedures

Patients must read and sign a current Ethics Committee (EC)-approved informed consent form before starting the study. All research steps need to be carried out within the time window indicated in the research schedule.

7.1 Screening period

After signing the informed consent, subjects entered the screening period. Unless otherwise specified, the following screening steps must be completed within 28 days prior to randomization:

- Demographic data: initials, gender, ethnicity, marital status, date of birth, height, weight;
- General medical history: past medical history and treatment history (clinical/pathological diagnosis, time of diagnosis, clinical/pathological stage, HER2/ER/PR/expression, whether surgery, whether neoadjuvant therapy, whether adjuvant therapy, whether radiotherapy, whether progressed to Recurrent/metastatic breast cancer), smoking and drinking history (frequency, amount, duration), drug allergy history (drug name, allergic symptoms), previous disease or concomitant disease/symptom (disease/symptom name, concomitant drug name, dosage, usage, outcome); bowel habits (frequency);
- Physical examination: including evaluation of the head, eyes, ears, nose, throat, skin, skeletal muscles, respiratory organs, cardiovascular, gastrointestinal, genitourinary and nervous systems. Anomalies at baseline are recorded in the CRF;
- Vital signs: including respiratory rate, pulse, systolic and diastolic blood pressure (subject is seated) and body temperature. Vital sign results should be obtained and assessed prior to each study treatment administration.
- KPS score;
- Blood routine: including hemoglobin, red blood cell count, platelet count, white blood cell count and neutrophil count.
- Blood biochemical tests: including sodium, potassium, chloride, cholesterol, triglyceride, glucose, BUN or urea, creatinine, total protein, albumin, alkaline phosphatase, ALT, AST, GGT, total bilirubin and direct bile Red and so on.
- Coagulation function test;
- Urine routine: including urine protein, urine sugar, and urine occult blood, check once each during the screening period and before surgery; if the urine routine shows urine protein ++ or above, please additionally check the 24-hour urine protein quantification;
- Stool routine: including fecal occult blood;
- Infectious disease screening: liver five items, HIV antibody, HCV antibody test;
- Pregnancy test: Female subjects of childbearing age should undergo blood HCG test during the screening period to exclude pregnancy;
- 12-lead ECG: need to check 3 times (at least 10 minutes apart), the average of the 3 QTc intervals will be used as the baseline QTc interval;
- Echocardiography: report within 28 days before randomization (including qualified echocardiography completed before signing ICF);
- Tumor hormone receptor status;
- Peripheral blood sample collection: set aside 8 mL of anticoagulant (intentional);
- Tumor histopathological sampling: During the screening period, tumor tissue samples were collected by needle biopsy. Each test center is responsible for collecting samples, and the Shengjing Hospital of China Medical

University will uniformly measure Ki67, [redacted] levels;

- Tumor imaging examination: including breast, thoracic, abdominal and bone scans. According to clinical conditions, imaging examinations of cranial brain, neck, pelvis and other parts can be added to exclude metastasis. CT or MRI can be used, and MRI is the choice for breast tumor evaluation. Investigators may add scan sites to baseline or subsequent tumor assessments as clinically warranted. Imaging results such as CT/MRI scans obtained prior to signing informed consent may be used for screening tumor assessment as long as they meet the requirements (and within 21 days prior to the first dose of study drug);
- Patient reported outcome (PRO): use EORTC QLQ-C30; version 3 (appendix V) and EORTC QLQ-BR23 ((appendix VI). Assessment should be performed at the baseline, the end of cycle2 and cycle 4, and before surgery. To avoid the influence of imaging examination results on the PRO assessment, the PROs should be assessed before imaging examination.
- Concomitant medication/treatment: record the concomitant medication from the screening period to the end of the study treatment;
- Adverse event follow-up: Recorded from the day the subjects signed the informed consent form, until at least 28 days after the last medication; after completing all the above screening evaluations, qualified persons began to receive trial drug treatment.

7.2 Treatment period

During the subjects receiving the trial drug treatment, the following inspection items should be completed within the time window listed in the trial process, and may be advanced accordingly in case of legal holidays. The first chemotherapy cycle will have 1 visit each on Day 7, Day 14, and Day 28, and then 1 visit at the end of each cycle (D28±3).

Investigators can increase the inspection items or increase the frequency of video visits according to the clinical conditions of the subjects:

- Vital signs: check once on the last day of each cycle during neoadjuvant therapy and before surgery;
- Blood routine: the 7th, 14th, and 28th days of the first cycle of neoadjuvant therapy, the last day of each subsequent cycle, and one check before surgery;
- Blood biochemical examination: on the 14th and 28th day of the first cycle of neoadjuvant therapy, on the last day of each cycle thereafter, and once before surgery;
- Coagulation function: 1 preoperative examination;
- Peripheral blood sample collection: 8 mL of anticoagulant was collected at 2 weeks of treatment, at the end of each cycle and before surgery (intentional);
- Tumor histopathological sampling: at 2 weeks of treatment (intentional), at the first efficacy evaluation (after the end of 2 cycles of treatment) at PD/SD (intentional), at the second efficacy evaluation (after the end of 4 cycles of treatment) PD /SD (intentional) by needle biopsy, at surgery by excision. Each test center is responsible for collecting samples and uniformly measuring Ki67 and apoptosis markers by Shengjing Hospital of China Medical University [redacted];
- 12-lead ECG: 1 examination at the end of each cycle; if the QTc interval increases by >30 msec from the baseline

during the test, or the absolute value of the QTc interval ≥ the specified value in any specified ECG measurement, 2 additional ECG examinations (at least 2) are required. interval of 10 minutes).

- Heart color Doppler ultrasound: During the study period, if symptoms such as chest pain and palpitations occur or the electrocardiogram indicates abnormality, additional investigations can be made as appropriate;
- Tumor imaging examination: The time point of tumor imaging examination during the dosing period is determined after the start of study treatment (i.e. C1D1), regardless of the time of drug suspension due to toxicity during this period. The time window allowed for tumor imaging was ±7 days at the end of cycle 2 and evaluation at the end of 4 cycles. Subjects discontinued trial treatment in the event of imaging-proven PD. No other anti-tumor therapy can be performed before PD occurs;
- Patient reported outcome (PRO): The time point of PROs during the dosing period is determined after the start of study treatment (i.e. C1D1), regardless of the time of drug suspension due to toxicity during this period. The time window allowed for tumor imaging was ±7 days at the end of cycle 2 and evaluation at the end of 4 cycles. Assessment will use EORTC QLQ-C30; version 3 (appendix V) and EORTC QLQ-BR23 ((appendix VI). To avoid the influence of imaging examination results on the PRO assessment, the PROs should be assessed before imaging examination.
- Concomitant medication/treatment: record information on concomitant medication/treatment during the study period at any time;
- Adverse events: observe and record adverse events during the study at any time;
- Disease progression/death: Observe and record disease progression and death time and specific conditions during the study period at any time.

7.3 Patient perioperative period

Subjects continued the medication until the patient underwent surgery. The following examinations should be performed and recorded in patients before surgery: physical examination, vital signs, KPS score, blood routine, blood biochemistry, peripheral blood sample collection, coagulation function test, urine routine, stool routine, pregnancy test, electrocardiogram, and tumor imaging evaluation and patient reported outcome.

Tumor samples were collected by excision during surgery. Each test center is responsible for collecting samples, which are uniformly determined by Shengjing Hospital of China Medical University Ki67, apoptosis marker [redacted] levels, check tpCR and RCB scores after surgery, and record them in real time Adverse events and concomitant medications.

7.4 End of study treatment/withdrawal from study

Subjects continued medication until completion of surgery, or disease progression, intolerable toxicity, withdrawal of knowledge, or discontinuation of medication at the discretion of the investigator. At the end of study treatment or withdrawal from the study, if the patient has not been tested within 7 days before the end of the study (except echocardiography, tumor imaging evaluation), the following tests should be performed:

- KPS score;
- vital signs;
- Physical examination;
- Blood routine: if not performed within 7 days prior;
- Urine routine: if not performed within 7 days prior ;
- Stool routine: if not performed within 7 days prior ;
- Blood biochemistry: if not performed within 7 days prior ;
- pregnancy test;
- 12-lead ECG: if not performed within 7 days prior ;
- Tumor imaging examination: if not performed within 8 weeks prior;
- Patient reported outcome (PRO): if not performed within 8 weeks prior;
- Concomitant medication/treatment: real-time recording;
- Adverse events: real-time recording;
- Drug recovery: recovery of the remaining drug in the test.

8 Effectiveness evaluation

8.1 Imaging assessment

Contrast-enhanced MRI is recommended for tumor imaging evaluation. Subjects with a history of contrast hypersensitivity were managed according to the trial center's guidelines for the prevention of contrast hypersensitivity reactions for enhanced MRI whenever possible. If the subject is strictly contraindicated to contrast media, color Doppler evaluation is allowed.

Imaging assessments will be performed according to the principles of the RECIST version 1.1 criteria. The screening period includes breast, chest, abdomen, and bone scans. According to clinical conditions, imaging examinations of the brain, neck, and pelvis can be added to exclude metastasis. Investigators may add scan sites to baseline or subsequent tumor assessments as clinically warranted. Imaging results such as CT/MRI scans obtained prior to signing informed consent may be used for screening tumor assessment as long as they are eligible (and within 21 days prior to the first dose of study drug).

Subsequent imaging assessments should be performed under the same conditions as the baseline examination (slice thickness of the scan, use of contrast agents, etc.). Tumor imaging time points during dosing were determined from the start of study treatment, and dosing suspensions did not change the time of evaluation. The allowable time window for tumor imaging examination is ±7 days, and the specific evaluation time points are as follows:

The first assessment will be conducted on the 28th day of the second cycle of dosing, and the second assessment will be conducted on the 28th day of the 4th cycle;

According to RECIST 1.1 criteria, bone scan and PET are not suitable for target lesion assessment. If necessary, if these tests are used to assess non-target lesions, the frequency of assessment of these non-target lesions can be reduced. For example, bone scans can be repeated only when CR is confirmed in target lesions or when bone lesions are suspected to have progressed.

8.2 Primary endpoint

Pathological Complete Response Rate (tpCR: ypT0-is/ypN0): Absence of any residual invasive carcinoma in hematoxylin- and eosin-stained resected breast cancer samples and all ipsilateral lymph node samples after completion of neoadjuvant therapy and surgery.

8.3 Secondary endpoints

Effectiveness Metrics:

1. Best Overall Response Rate (BORR): The proportion of patients who achieved a response at any point during the study

2. bpCR: ypT0-is

3. Residual tumor burden (RCB)

4. Changes in molecular targets from baseline to surgery:

(1) Ki67 apoptosis marker

[redacted]

Safety Indicators:

Adverse Events (AEs) and Serious Adverse Events (SAEs). Refer to NCI-CTC AE 5.0 standard.

Exploratory research:

The Patient-Reported Outcome (PRO) data will be scored according to the EORTC scoring manual using the EORTC QLQ-C30 version 3) and the EORTC-QLQ-BR23, which included different dimensions (global health status, functional scales, symptom scales and single items). A raw score was calculated as the average of items contributing to a scale and standardized to a range of 0 to 100 points. On HRQoL and functioning scales a higher score indicated better status. Higher scores on symptom scales indicated worse symptoms. The changes in scores for different HRQoL dimensions from baseline to every timepoints will be analyze.

Anticoagulant 8ml at baseline, at 2 weeks of treatment, at the end of each cycle, and at the time of surgery were collected and saved (intention-to-treat) for an exploratory study of factors that may affect or predict efficacy. Safety indicators: exposure to the investigational drug and the incidence, nature and severity of adverse events (including serious adverse events).

9 Safety evaluation

During the trial period, the safety of the study drug was evaluated through adverse event records (including serious adverse events), laboratory tests, vital signs, physical examination, KPS score, echocardiography and electrocardiogram records. During the trial, the symptoms and signs of the subjects after the drug should be closely observed. Adverse events/reactions that occur should be dealt with in a timely and effective manner to ensure the safety and interests of the subjects. After the occurrence of adverse drug events/reactions has been dealt with in a timely and effective manner, the type, symptoms, time of occurrence, degree (or grade), symptomatic treatment methods and outcomes should be recorded, and then the adverse events should be analyzed, assessed, and counted as the basis for the continuation of the test.

9.1 Physical examination and vital signs

The physical examination is carried out by the research doctor, and the examination contents include: general condition, skin and mucous membranes, lymph nodes, head and neck, chest, abdomen, musculoskeletal, neural reflex, respiratory system, cardiovascular system, genitourinary system, mental condition, etc.

Vital signs include the following: including respiratory rate, pulse, heart rate, blood pressure and temperature.

The KPS score was scored by the study physician according to the KPS scoring scale.

9.2 laboratory test

The following laboratory indicators will be tested according to the time points specified in the "Clinical Trial Flowchart". Unscheduled clinical laboratory tests may be performed at any time due to subject safety concerns.

Table 3 Content requirements for laboratory tests

Blood routine	Blood chemistry	Urine routine
hemoglobin<br>red blood cells leukocyte<br>Neutrophil count lymphocyte count platelet count	total bilirubin<br>Direct bilirubin<br>ALT<br>AST<br>alkaline phosphatase<br>r-GT<br>Total protein<br>albumin<br>urea nitrogen<br>uric acid<br>Creatinine<br>blood sugar<br>Potassium<br>sodium<br>chlorine<br>calcium<br>phosphorus<br>magnesium	Urine protein [a]<br>Urine sugar<br>Urine red blood cells<br>Urinary white blood cells
Coagulation	Infectious Disease Screening	other
INR<br>APTT<br>PT FIB<br>TT	Hepatitis B five items<br>HIV antibodies<br>HCV antibodies	pregnancy test [b]

notes：

a. If the semi-quantitative method shows urine protein ++ or above, perform a 24-hour urine protein quantification test.

b. Females of childbearing age should undergo blood HCG testing to exclude pregnancy during the screening period, and urine HCG testing can be performed at other time points.

9.3 ECG

The 12-lead ECG will be performed by a qualified physician according to the time points specified in the clinical trial flow chart.

All ECG examinations are required to be performed after the subject has rested in a quiet recumbent position for at least 10 minutes. Contents include at least: heart rate, QT, QTc and P-R time. During the screening period, three examinations (at least 10 minutes apart) are required, and the average of the three QTcFs will be used as the baseline QTcF.

To assess subject safety, study physicians will compare ECG results to baseline testing. If the QTc interval increases by >30 msec from the baseline, or the absolute value of the QTc interval is greater than or equal to the specified value in any of the specified ECG measurements, 2 additional ECG examinations are required, with an interval of at least 10 minutes, to determine the accuracy of the original measurement and exclude the incorrect cause. The routing of the leads results in an abnormal ECG. If the machine-read QTc value is prolonged, repeat measurements may not be performed if a qualified physician determines that the QTc value is within an acceptable range, as described above.

9.4 Echocardiography

Cardiac ultrasound will be performed by a qualified physician according to the time points specified in the clinical trial flow chart. LVEF will be assessed and monitored by the study physician at protocol-specified time points during administration of the investigational drug. During the administration process, according to the judgment of the investigator, subjects with symptoms of heart failure or clinically significant LVEF decline should be treated and monitored in accordance with standard medical guidelines, and a cardiologist should be consulted if necessary. In the event of clinically uncontrollable symptoms of severe heart failure (NYHA class III or IV) or a significant decrease in LVEF (below the lower limit of normal or below 50%), administration of the trial drug should be discontinued as specified in Section 5.4 - Dose Adjustment Protocol medication, and continue to be treated and monitored according to standard medical guidelines.

9.5 Adverse event (AE）

9.5.1 Definition of Adverse Events

Adverse events refer to any adverse medical events that occur after the subjects of the clinical trial signed the informed consent, but the events do not necessarily have a causal relationship with the study drug. An adverse event can be any unexpected, unfavorable symptom, sign, disease, or abnormal examination result, whether related to the study drug or not. Adverse events include the following: 1) a medical condition/disease that existed prior to initiation of study treatment, and was only recorded as an adverse event if it worsened after initiation of study drug; 2) any new adverse event; 3) experimental Abnormal changes in laboratory findings that are considered clinically significant constitute an adverse event.

9.5.2 Adverse Event Severity Judgment Criteria

Refer to the NCI-CTC AE 5.0 grading criteria for adverse drug events.

In the event of adverse events not listed in the NCI-CTC AE version 5.0 table, the following criteria can be used:

Grade I: Mild; no clinical symptoms or mild clinical symptoms; clinical or diagnostic findings only; no treatment required.

Grade II: Moderate; minimal, topical, or non-invasive treatment required; age-appropriate instrumental activities of daily living (IADL) limitations. Instrumental activities of daily living include cooking, shopping, making phone calls, managing money, etc.

Class III: Severe or medically significant but not immediately life-threatening; causes hospitalization or prolonged hospitalization; causes disability; limits self-care activities of daily living (Self-care ADL). Self-care activities of daily living refer to: bathing, dressing, undressing, eating, going to the toilet, taking medicine, etc., non-bedridden.

Class IV: Life-threatening consequences; urgent medical attention required.

Grade V: Deaths related to adverse events.

9.5.3 Judgment criteria for the relationship between adverse events and the investigational drug

Adverse events include all unexpected clinical manifestations, as long as these events occur after signing the informed consent form, regardless of whether they are related to the test drug, whether they are assigned to the test drug group, or even whether the drug is used or not, they should be reported as adverse events. Any discomfort reported by the patient during the treatment of the test case or abnormal changes in the objective laboratory test indicators should be recorded truthfully, and the severity, duration, treatment measures and outcome of the adverse event should be indicated at the same time. To determine the relationship between adverse events and the trial drug, the possible association between adverse events and the trial drug was evaluated according to the five-level classification method of "definitely related, possibly related, possibly irrelevant, definitely irrelevant, and unable to determine".

"Definitely related", "possibly related" and "undeterminable" are listed as adverse drug reactions, and the total of the two is used as the numerator when calculating the incidence of adverse events, and the total number of subjects used to evaluate safety is used as the denominator. The judgment criteria are shown in Table 4:

Table 4 Judgment criteria for the relationship between adverse events and the investigational drug

Grading	Judgement standard
Definitely related	The occurrence of the events conforms to a reasonable time sequence after the drug is administered, the event conforms to the known reaction type of the suspected drug, improves after the drug is discontinued, and the event occurs again after repeated administration.
Possibly related	The occurrence of the event conforms to a reasonable time sequence after the drug is administered, the event does not conform to the known response type of the drug in question, and the clinical status of the patient or other treatment modalities may also produce the event.
Unlikely related	The occurrence of the event does not conform to a reasonable chronological sequence after the drug is administered, the event does not conform to the known response type of the suspected drug, and the clinical status of the patient or other treatment modalities may also produce the event.
Definitely unrelated	The occurrence of the event does not conform to a reasonable chronological sequence after the drug, the event does not conform to the known response type of the suspected drug, the patient's clinical state or other treatment methods may also produce the event,

	the disease is improved or the event is eliminated after other treatment methods are discontinued, and repeated use Other therapy events occurred.
Not Assessable	The occurrence of events is not clearly related to the chronological sequence after the drug is used, and it is similar to the known response type of the drug, and other drugs used at the same time may also cause the corresponding event.

9.5.4 Recording and reporting of adverse events

Investigators should record any adverse events that occur in subjects in detail, including: description of adverse events and all related symptoms, time of occurrence, severity, duration, measures taken and final outcome (recovery/cure, presence or absence of sequelae, remission, no change, death, unknown). The trial begins on the day the subjects signed the informed consent form and needs to be evaluated for drug safety until 28 days after the last dose.

All adverse events (serious and non-serious) should be recorded on the adverse event reporting page of the case report form, and adverse events should be reported using accurate medical terminology.

Related AEs must be followed until any one of the following occurs:

1) Disappeared or return to baseline levels;
2) Reassessment considered unrelated to the investigational drug (pyrotinib, SHR6390, letrozole and/or goserelin);
3) Death;
4) Start a new anti-tumor treatment regimen;
5) The investigator confirmed that no further improvement is expected and that the patient's condition is stable;
6) Clinical data or final databases are no longer collected.

Unrelated AEs must be followed until any one of the following occurs :

1) disappeared or improved to the baseline level;
2) The severity is improved to within grade 1;
3) death;
4) Start a new anti-tumor treatment regimen;
5) The investigator confirms that no further improvement is expected;
6) No more clinical data or final database collection.

9.6 Serious adverse event（SAE）

9.6.1 Definition of Serious Adverse Events

Serious adverse events (SAEs) refer to medical events that require hospitalization or prolonged hospitalization, disability, affect work ability, endanger life or death, and cause congenital deformities during clinical trials. Adverse events that meet one or more of the following criteria are SAEs:

✓ Results in death;

✓ Life-threatening (defined as a subject in immediate danger of death at the time of the event);

✓ Results in hospitalization or prolonged hospitalization;

✓ Results permanent or severe disability/incapacity;

✓ Results in congenital anomalies or birth defects;

✓ Important Medical Events: These adverse events may not result in death, life threatening, or require hospitalization, but in medical judgment, events may harm the subject and require medical or surgical intervention to prevent any of the above outcomes.

Investigators should report all serious adverse events in a timely manner, including clinical diagnosis and treatment and outcomes, and follow up until they return to normal, remission or stability. At the same time, make detailed records in the original medical records and fill in the SAE report form.

9.6.2 Potential drug-induced liver injury

If the ALT and/or AST levels are abnormal and the total bilirubin level is abnormally elevated, the following conditions are met and there is no other cause of liver injury, it will be considered as drug-induced liver injury. Such conditions should always be considered a medically important event.

Table 5 Potential Drug-Induced Liver Injury Evaluation Criteria

<table>
<tr><td>Baseline</td><td colspan="2">Normal (ALT/AST and total bilirubin)</td><td colspan="2">Abnormal (ALT/AST and total bilirubin)</td></tr>
<tr><td rowspan="2">Treatment period</td><td>ALT≥3×ULN</td><td>AST≥3×ULN</td><td>ALT or AST ≥ 2 x baseline level and value ≥ 3 x ULN</td><td>ALT or AST≥8×ULN</td></tr>
<tr><td colspan="2">● meet either of the above
● With total bilirubin≥2×ULN
● And alkaline phosphatase≤2×ULN
● without hemolysis</td><td colspan="2">● meet either of the above
● And with total bilirubin ≥1×ULN or value ≥3×ULN</td></tr>
</table>

Subjects should return to the study center for evaluation as soon as possible (preferably within 48 hours) after learning of an abnormal result. Evaluation should include laboratory tests, a detailed history, and physical evaluation, and should consider the possibility of liver tumors (primary or secondary).

In addition to repeat testing of ALT and AST, laboratory tests to be performed should include albumin, creatine kinase, total bilirubin, direct and indirect bilirubin, gamma glutamyl transferase, prothrombin time. [PT])/international normalized ratio (INR), alkaline phosphatase. Further workup may also include testing for acute hepatitis A, B, C, and E and liver imaging.

A detailed medical history should include: alcohol consumption, acetaminophen, soft drugs, various supplements, family medical history, occupational exposure, sexual behavior, travel history, contact with a patient with jaundice, surgery, blood transfusion, liver disease or History of allergic diseases, etc.

If repeat testing still confirms compliance with the definitions of the above laboratory criteria, in the absence of other causes of abnormal liver function tests, the possibility of underlying drug-induced liver injury should be considered without waiting for all liver function etiology tests to be performed result. Such cases of potential drug-induced liver injury should

be reported as SAEs.

9.6.3 Disease progression

Disease progression (including signs and symptoms of progression) should not be reported as a serious adverse event, but death due to disease progression should be reported within the trial or safety reporting period, i.e. within 28 days (inclusive) of the last dose of study drug Serious adverse events. Hospitalizations due to symptoms and signs of disease progression should not be reported as serious adverse events. During the trial or safety reporting period, if the final outcome of cancer is death, the event leading to death must be reported as a serious adverse event.

9.6.4 Hospitalization

Adverse events leading to hospitalization or prolonged hospital stay in clinical studies should be considered serious adverse events. Any initial admission to a medical facility (even if it is less than 24 hours) meets this criterion.

The following hospitalizations do not constitute an SAE:

- ✓ Rehabilitation institutions
- ✓ Nursing home
- ✓ Routine emergency room admissions
- ✓ Same-day surgery (e.g., outpatient/same-day/ambulatory surgery)
- ✓ Hospitalization or prolonged hospital stay not related to adverse events are not serious adverse events in themselves. For example:
- ✓ Admission due to pre-existing disease, no new adverse events occurred, and no exacerbation of pre-existing disease (e.g., to check for laboratory abnormalities that persisted before the trial);
- ✓ Manage hospitalizations for reasons (e.g., routine annual physicals);
- ✓ Hospitalization stipulated in the trial protocol during the clinical trial (for example: operating according to the requirements of the trial protocol);
- ✓ Elective hospitalization unrelated to adverse events (e.g. elective cosmetic surgery);
- ✓ Scheduled treatments or surgical procedures should be recorded in the overall trial protocol and/or in the individual subject's baseline data;
- ✓ Hospital admission only for blood product use.

Diagnostic or therapeutic invasive (e.g., surgery), non-invasive procedures should not be reported as adverse events. However, the medical condition leading to the procedure should be reported when it meets the definition of an adverse event, e.g., acute appendicitis that developed during the reporting period should be reported as an adverse event, and appendectomy performed as a result should be recorded as the treatment for that adverse event.

9.6.5 Serious Adverse Event Reporting System

The reporting of serious adverse events should begin with the subject signing the informed consent form until the end of the safety follow-up period. In the event of a serious adverse event, whether it is the first report or the follow-up report, the investigator must immediately fill out the "Serious Adverse Event (SAE) Report Form", sign and date it, and immediately notify the principal investigator within 24 hours after the investigator is informed of the SAE. unit and Hengrui drug safety department, and report to relevant units in a timely manner according to regulatory requirements (see Table 6. SAE reporting method).

SAEs that occur after the safety follow-up period should be collected for those suspected of being related to the study drug. Serious adverse events should be recorded in detail with symptoms, severity, correlation with the test drug, time of occurrence, treatment time, measures taken, follow-up time and method, and outcomes. If the investigator believes that a serious adverse event is not related to the investigational drug, but is potentially related to a study condition (eg, discontinuation of the original treatment, or comorbidities during the trial), this relationship should be detailed in the narrative section of the SAE report form. If there is a change in the intensity of an ongoing serious adverse event or its relationship to the test drug, a follow-up report should be submitted immediately. If the investigator believes that the previously reported SAE has misreported information, it can be corrected, withdrawn or downgraded in the follow-up report and reported according to the SAE reporting procedure.

The email address of Hengrui Drug Safety Department for receiving SAE reports in this project is: hengrui_drug_safety@hrglobe.cn.

Table 6. SAE reporting method

unit	contact	Fax/Telephone/Address
Shengjing Hospital Affiliated to China Medical University	Ethics Committee	[redacted]
Jiangsu Hengrui Pharmaceutical Co., Ltd.	Drug safety department	[redacted]
State Drug Administration Department of Drug Registration		[redacted]

National Health Commission Medical Administration and Hospital Administration Medical Safety and Blood Division		[redacted]
Provincial, autonomous region and municipal drug administration departments	Refer to the reporting requirements of the drug regulatory authorities of the provinces, autonomous regions and municipalities directly under the Central Government	

9.7 Pregnancy

If a female subject becomes pregnant during the clinical trial, the subject is out of the group.

Investigators were required to follow up on pregnancy outcomes until 1 month after the mother gave birth.

If a subject experiences SAE during pregnancy, the NMPA Serious Adverse Event Reporting Form then needs to be filled out, and reported according to SAE reporting procedure.

If the pregnancy result is stillbirth, spontaneous miscarriage, or fetal malformation, it is considered as SAE, and the NMPA Serious Adverse Event Reporting Form needs to be filled out, and it needs to be reported according to the time limit and requirements of SAE.

10 Research management

10.1 Ethics and Informed Consent

10.1.1 Code of Ethics

This clinical trial must comply with the Declaration of Helsinki (2008 edition), the Good Clinical Practice for Drugs (GCP) promulgated by the NMPA, and related regulations. Prior to the start of the trial, approval from the unit's ethics committee must be obtained before the study can begin. During the clinical research period, any modification of the trial protocol should be reported to the ethics committee and put on record. The principal investigator is responsible for regularly submitting the interim report of the trial in accordance with the relevant requirements of the ethics committee, and should notify the ethics committee that the trial has ended after the trial is over.

10.1.2 Informed consent

Subjects must give informed consent to participate in this trial before receiving the drug in this trial, in order to protect the legitimate rights and interests of the subjects. The researcher is responsible for fully and comprehensively introducing the purpose of this study, the role of the drug, possible toxic and side effects and possible risks to the subjects or their designated representatives. risks and benefits. Conversation is a very important informed consent process. If the subjects and their legal representatives are illiterate, witnesses should participate in the informed consent process. After the subjects or their legal representatives give oral consent, they should sign the informed consent form. Signed on the same day. The informed consent form should indicate the version number and version date.

10.2 Amendment of the plan

Any necessary changes to the protocol must be made in the form of protocol revisions, and must be submitted to the ethics committee for approval or filing after the principal investigator's signature and approval, and the details of previous revisions should be explained in the protocol.

10.3 Data management

10.3.1 Data collection

In this study, CRF was used for the collection of research data; the personnel of the research institution were required to carry out systematic training.

10.3.2 Data Management and Quality Control

In order to ensure the authenticity and reliability of clinical trial data and improve the quality of clinical data, the clinical monitor will review the integrity, consistency and accuracy of the trial data in the clinical database in accordance with standard operating procedures during the course of the trial project, and provide guidance. The personnel of the research institution shall make necessary additions or corrections to the question data. If clinical personnel or data management personnel have questions about the data, they need to question the researchers or data entry personnel. The relevant personnel must respond to the questioning and make corrections or explanations to the question data. Problem data solved. Medical staff and data managers regularly perform consistent comparisons of SAEs.

At the end of the trial project, data managers and medical staff will conduct final quality control of all data, summarize all protocol deviations and protocol violations that occurred during the trial, and hold a data verification meeting. Research statisticians can conduct data analysis only after all data meet the quality requirements.

10.3.3 Data review and monitoring of research institutions

Before the start of the trial, the research leader needs to introduce the trial protocol and CRF. During the trial, the person in charge of data monitoring shall regularly check the integrity of the patient records and the accuracy of the content on the CRF, compliance with the trial protocol and drug clinical trial quality management practices, progress of enrollment, and ensure that the trial drugs are stored in accordance with regulations. , distribution and counting. During this period, key researchers must be able to assist data monitors.

Investigators must maintain original documentation for each patient enrolled in the trial, including study medical records and visit records (inpatient or outpatient medical records) that include demographic indicators and medical information, laboratory data, electrocardiograms, and any other examinations or evaluations the result of. All information on the CRF must be derived from the original documents in the patient file. The investigator must also keep the informed consent form signed by the patient. Investigators must verify that all relevant source documents can be reviewed to verify that they are consistent with the CRF content. Monitoring criteria require 100% monitoring of informed consent obtained, compliance with inclusion/exclusion criteria, documentation of SAEs, and data required for evaluation of all primary and safety measures. Additional checks for consistency of raw data and CRF were performed in accordance with the trial-specified monitoring plan. Any information on the patient's

identity in the original file will not be made public.

10.4 Plan violation

All requirements specified in the research protocol must be strictly implemented. Any intentional or unintentional deviation or violation of the protocol and GCP principles can be classified as a protocol deviation or protocol violation. If any deviation from the protocol is found, the investigator should fill in the protocol violation record, record in detail the time of discovery, the time and process of the incident, the reason and the corresponding treatment measures, signed by the principal investigator, and notified to the ethics committee.

11 Data Analysis and Statistical Methods

11.1 Sample size calculation

The study estimated the sample size based on the primary endpoint pCR rate. According to literature reports, the pCR rate of anti-HER2 single target (trastuzumab) combined with endocrine therapy for neoadjuvant treatment of breast cancer is about 15%. Assuming the pCR rate of the treatment regimen achieves 26% [the rate of anti-HER2 dual target (trastuzumab/pertuzumab) combined with chemotherapy]. The sample size calculation was performed using the simon two-stage design scheme with one-sided test, 5% type I error, and an 80% power. It is calculated that 61 cases are needed to be enrolled in the first stage. Considering a dropout rate of 10%, 67 cases will be enrolled in the first stage. If 12 or more pCR were noted, recruitment proceed for an additional 20 evaluable patients. If 18 or more of 81 patients achieved pCR, the study treatment would be deemed worthy of future study.

11.2 Statistical Analysis Program

11.2.1 Statistical analysis dataset

The analysis population of this study included Full Analysis Set (FAS), Per Protocol Set (PPS) and Safety Set (SS).

Full Analysis Set (FAS): According to the mITT principle, include patients who received at least one cycle of the combination, exclud patients who were withdrawn due to their subjective factors.

Per-protocol set (PPS): A subset of FAS, patients who met the study protocol, completed 5 cycles of neoadjuvant therapy and surgery.

The efficacy analysis of the trial drug was performed for the full analysis set and the protocol-compliant set.

Safety Set (SS): All enrolled subjects with at least one medication record will be included in the safety set. The safety analysis of this trial drug was performed on the safety analysis set.

11.2.2 Statistical analysis methods

11.2.2.1 Basic method

In this study, unless otherwise specified, the data will be summarized using descriptive statistics according to the following general principles.

Measurement data were summarized using mean, standard deviation, median, maximum value, and minimum

value; count data were summarized by frequency and percentage, and 95% confidence interval of percentage was given if necessary; time-event data were estimated by Kaplan-Meier Survival rates and plot survival curves.

11.2.2.2 Efficacy Endpoint Analysis

The primary endpoint, pCR rate, will be analyzed based on the FAS and PPS sets, with FAS being the primary analysis set.

Descriptive analysis of pathological complete response rate (pCR); best overall response rate (BORR); residual tumor burden (RCB); changes in molecular targets from baseline to surgery: ①Ki67 apoptotic marker, [redacted];

5 years DFS; 5 years OS.

11.2.2.3 Security Analysis

All adverse events (AEs) will be coded using MedDRA and graded according to the NCI CTC AE version 5.0 grading system. A treatment-emergent adverse event (TEAE) was defined as any adverse event that was new or worse than baseline (before study treatment) after initiation of study drug.

The safety analysis will be dominated by descriptive statistical summaries. For AEs, SAEs, AEs of grade ≥3, SAEs of grade ≥3, drug-related

Data on AEs, drug-related SAEs, AEs with an incidence of ≥5%, SAEs with an incidence of ≥5%, AEs leading to dose adjustment, and AEs leading to discontinuation of treatment were statistically pooled.

Describe the laboratory test results that were normal before the test but abnormal after treatment, and the relationship to the test drug when abnormal changes occurred.

Differences in vital signs from baseline were summarized by visit; baseline and highest post-baseline KPS scores were summarized; and baseline and post-baseline worst clinical abnormality grades were summarized by ECG and echocardiography.

Describe the laboratory test results that were normal before the test but abnormal after treatment, and the relationship to the test drug when abnormal changes occurred.

Differences in vital signs from baseline were summarized by visit; baseline and highest post-baseline KPS scores were summarized; and baseline and post-baseline worst clinical abnormality grades were summarized by ECG and echocardiography.

Security analysis includes, but is not limited to, the above analysis.

11.2.2.4 PRO Analysis

The EORTC QLQ-C30 included global health status, functional scales (physical, role, emotional, cognitive, and social), symptom scales (fatigue, nausea/vomiting, and pain) and single items (dyspnoea, insomnia, appetite loss, constipation, diarrhea, and financial difficulties). Most questions used a 4-point scale (1 'Not at all' to 4 'Very much'; 2 questions used 7-point scale [1 'very poor' to 7 'Excellent']). Scores were averaged and transformed to 0 - 100 scale,

whereby higher scores indicate greater functioning, greater quality of life, or a greater degree of symptoms, with changes of 5 - 10 points considered to be a minimally important difference to participants. A positive value means an increase, while a negative value means a decrease, in score at the indicated time-point relative to the score at baseline.

EORTC-QLQ-BR23 is a 23-item breast cancer-specific companion module to the EORTC-QLQ-C30 and consists of four functional scales (body image, sexual enjoyment, sexual functioning, future perspective [FP]) and four symptom scales (systemic side effects [SE], upset by hair loss, arm symptoms, breast symptoms). Questions used 4-point scale (1=not at all, 2=a little, 3=quite a bit, 4=very much). Scores averaged and transformed to 0-100 scale. High score for functional scale indicated high/better level of functioning/healthy functioning. Higher scores for symptom scales represent higher levels of symptoms/problems. For functional scales, positive change from baseline indicated deterioration in quality of life (QOL) and negative change from baseline indicated an improvement in QOL. For symptom scales, positive change from baseline indicated an improvement in quality of life (QOL) and negative change from baseline indicated a deterioration in QOL.

The changes in scores for different HRQoL dimensions from baseline to every timepoints will be analyze.

11.2.3Statistics Software

SAS9.4 or above were used for analysis.

12 Case dropout

All subjects who have filled out the informed consent form and are screened to be eligible to enter the trial have the right to withdraw from the clinical trial at any time. No matter when and for any reason, as long as the subjects who have not completed at least one trial drug administration and cannot be evaluated for safety and efficacy, they are dropout cases (disregarded due to disease progression and clear medical evidence after enrollment). to fall off). When a subject drops out, the researcher must record the reason for the drop out, complete the assessment items that can be completed, and fill in the last visit record carefully. For those who fall off due to adverse reactions and are finally judged to be related to the trial drug after follow-up, the investigator should be notified. Subjects who dropped out of the study after screening without obtaining a drug number were not considered dropout cases. If subjects completed a complete cycle and have detailed records, statistical analysis should be performed in the safety evaluation.

Subjects who withdraw from the study cannot be re-entered and their number cannot be reused.

Appendix I: Clinical Staging Criteria for Breast Cancer (Eighth Edition AJCC Breast Cancer TNM Staging）

Stage 0	TisN0M0
Stage I	T1N0M0
Stage IIA	T0N1M0
	T1N1M0
	T2N0M0
Stage IIB	T2N1M0
	T3N0M0
Stage IIIA	T0N2M0
	T1N2M0
	T2N2M0
	T3N1、2M0
Stage IIIB	T4N0M0，T4N1M0，T4N2M0
Stage IIIC	Any T，N3M0
Stage IV	Any T Any N，M1

Appendix II：Karnofsky Performance Status Scale（KPS）

Condition	Level of functional capacity	Value (%)
Able to carry on normal activity and to work. No special care needed.	No complaints; no evidence of disease	100 %
	Able to carry on normal activity; minor signs or symptoms of disease	90 %
	Normal activity with effort; some signs or symptoms of disease	80 %
Unable to work; able to live at home and care for most personal needs; varying amount of assistance needed	Cares for self; unable to carry on normal activity or to do active work	70 %
	Requires occasional assistance but is able to care for most personal needs	60 %
	Requires considerable assistance and frequent medical care	50 %
Unable to care for self; requires equivalent of institutional or hospital care; diseases may be progressing rapidly.	Disabled; requires special care and assistance	40 %
	Severely disabled; hospital admission indicated although death not imminent	30 %
	Very sick; hospital admission necessary; active supportive treatment necessary	20 %
	Moribund; fatal processes progressing rapidly	10 %
	Dead	0 %

Appendix III：2018 ASCO/CAP Breast Cancer HER2 Testing Guidelines

Topic	2018 ASCO/CAP Breast Cancer HER2 Testing Guidelines	
HER2 testing<br>(invasive component)<br>by validated<br>IHC assay	IHC 0 negative	No staining is observed or Membrane staining that is incomplete and is faint/barely perceptible and in ≤ 10% of tumor cells
	IHC 1+ negative	Incomplete membrane staining that is faint/barely perceptible and in > 10% of tumor cells
	IHC 2+ equivocal	Weak to moderate complete membrane staining observed in > 10% of tumor cells
	IHC 3+ positive	Circumferential membrane staining that is complete, intense, and in > 10% of tumor cells[1]
HER2 testing<br>(invasive component)<br>by validated<br>dual-probe ISH assay	HER2/CEP17 ratio ≧ 2.0<br>Average HER2 copy number≧ 4.0 signals/cell	ISH positive
	HER2/CEP17 ratio ≧ 2.0<br>Average HER2 copy number＜ 4.0 signals/cell	Assess IHC using sections from the same tissue sample used for ISH：<br>IHC 0 or 1＋，HER2 negative with comment[2]<br>IHC 2＋，Observer blinded to previous results recounts ISH, counting at least 20 cells, if HER2/CEP17 Ratio ≥ 2.0 and Average HER2 signals/cell ＜ 4.0, HER2 negative with comment*; Other ISH result, Result should be adjudicated per internal procedures to determine final category<br>IHC 3＋，HER2 positive
	HER2/CEP17 ratio < 2.0<br>Average HER2 copy number≧ 6.0 signals/cell	Assess IHC using sections from the same tissue sample used for ISH：<br>IHC 0 or 1＋，HER2 negative with comment[3]<br>IHC 2＋，Observer blinded to previous results recounts ISH, counting at least 20 cells, if HER2/CEP17 Ratio ＜ 2.0 and Average HER2 signals/cell ≥ 6.0, HER2 positive; Other ISH result, Result should be adjudicated per internal procedures to determine final category<br>IHC 3＋，HER2 positive
	HER2/CEP17 ratio < 2.0<br>Average HER2 copy number≧ 4.0 and ＜ 6.0 signals/cell	Assess IHC using sections from the same tissue sample used for ISH：<br>IHC 0 or 1＋，HER2 negative with comment[4]<br>IHC 2＋，Observer blinded to previous results recounts ISH, counting at least 20 cells, if HER2/CEP17 Ratio ＜ 2.0 and Average HER2 signals/cell ≥ 4.0 and ＜ 6.0, HER2 negative with comment[b]; Other ISH result, Result should be adjudicated per internal procedures to determine final category<br>IHC 3＋，HER2 positive
	HER2/CEP17 ratio <2.0<br>Average HER2 copy number ＜ 4.0 signals/cell	ISH negative

Note:

(1) Readily appreciated using a low power objective and observed within a homogeneous and contiguous invasive cell population.

(2) Evidence is limited on the efficacy of HER2-targeted therapy in the small subset of cases with a HER2/CEP17 ratio ≥ 2.0 and an average HER2 copy number of ＜ 4.0 per cell. In the first generation of adjuvant trastuzumab trials, patients in this subgroup who were randomly assigned to the trastuzumab arm did not seem to derive an improvement in disease-free or overall survival, but there were too few such cases to draw definitive conclusions. IHC expression for HER2 should be used to complement ISH and define HER2 status. If the IHC result is not 3+ positive, it is recommended that the specimen be considered HER2 negative because of the low HER2 copy number by ISH and the lack of protein overexpression CEP17, chromosome enumeration probe 17; HER2, human epidermal growth factor receptor 2; IHC, immunohistochemistry; ISH, in situ hybridization

(3) There are insufficient data on the efficacy of HER2-targeted therapy in cases with a HER2 ratio of ＜ 2.0 in the absence of protein overexpression because such patients were not eligible for the first generation of adjuvant trastuzumab clinical trials. When concurrent IHC results are negative (0 or 1+), it is recommended that the specimen be considered HER2 negative. CEP17, chromosome enumeration probe 17; HER2, human epidermal growth factor receptor 2; IHC, immunohistochemistry; ISH, in situ hybridization

(4)) It is uncertain whether patients with an average of ≥ 4.0 and ＜ 6.0 HER2 signals per cell and a HER2/CEP17 ratio of ＜ 2.0 benefit from HER2-targeted therapy in the absence of protein overexpression (IHC 3+). If the specimen test result is close to the ISH ratio threshold for positive, there is a higher likelihood that repeat testing will result in different results by chance alone. Therefore, when IHC results are not 3+ positive, it is recommended that the sample be considered HER2 negative without additional testing on the same specimen. CEP17, chromosome enumeration probe 17; HER2, human epidermal growth factor receptor 2; IHC, immunohistochemistry; ISH, in situ hybridization.

Appendix IV: New response evaluation criteria in solid tumors: Revised RECIST guideline

New response evaluation criteria in solid tumours: Revised RECIST guideline (version 1.1)（excerpts）

Note: This attachment is an internal translation material for reference only, the English version shall prevail in actual operation.

1. Background
2. Purpose
3. Measurability of tumour at baseline

3.1. Definitions

At baseline, tumour lesions/lymph nodes will be categorized measurable or non-measurable as follows:

3.1.1. Measurable

Tumour lesions: Must be accurately measured in at least one dimension (longest diameter in the plane of measurement is to be recorded) with a minimum size of:

- 10 mm by CT scan (CT scan slice thickness no greater than 5 mm; see Appendix II on imaging guidance).
- 10 mm caliper measurement by clinical exam (lesions which cannot be accurately measured with calipers should be recorded as non-measurable).
- 20 mm by chest X-ray.

Malignant lymph nodes: To be considered pathologically enlarged and measurable, a lymph node must be P15 mm in short axis when assessed by CT scan (CT scan slice thickness recommended to be no greater than 5 mm). At baseline and in follow-up, only the short axis will be measured and followed (see Schwartz et al. in this Special Issue15). See also notes below on 'Baseline documentation of target and non-target lesions' for information on lymph node measurement.

3.1.2. Non-measurable

All other lesions, including small lesions (longest diameter <10 mm or pathological lymph nodes with P10 to <15 mm short axis) as well as truly non-measurable lesions. Lesions considered truly non-measurable include: leptomeningeal disease, ascites, pleural or pericardial effusion, inflammatory breast disease, lymphangitic involvement of skin or lung, abdominal masses/abdominal organomegaly identified by physical exam that is not measurable by reproducible imaging techniques.

3.1.3. Special considerations regarding lesion measurability, Bone lesions, cystic lesions, and lesions previously treated with local therapy require particular comment:

Bone lesions:

- Bone scan, PET scan or plain films are not considered adequate imaging techniques to measure bone lesions. However, these techniques can be used to confirm the presence or disappearance of bone lesions.
- Lytic bone lesions or mixed lytic-blastic lesions, with identifiable soft tissue components, that can be evaluated by cross sectional imaging techniques such as CT or MRI can be considered as measurable lesions if the soft tissue component meets the definition of measurability described above. of measurability described above.
- Blastic bone lesions are non-measurable.

Cystic lesions:

- Lesions that meet the criteria for radiographically defined simple cysts should not be considered as malignant lesions (neither measurable nor non-measurable) since they are, by definition, simple cysts.
- 'Cystic lesions' thought to represent cystic metastases can be considered as measurable lesions, if they meet the definition of measurability described above. However, if non-cystic lesions are present in the same patient, these are preferred for selection as target lesions.

Lesions with prior local treatment:

- Tumour lesions situated in a previously irradiated area, or in an area subjected to other loco-regional therapy, are usually not considered measurable unless there has been demonstrated progression in the lesion. Study protocols should detail the conditions under which such lesions would be considered measurable.

3.2. Specifications by methods of measurements

3.2.1 Measurement of lesions

All measurements should be recorded in metric notation, using calipers if clinically assessed. All baseline evaluations should be performed as close as possible to the treatment start and never more than 4 weeks before the beginning of the treatment.

3.2.2 Method of assessment

The same method of assessment and the same technique should be used to characterize each identified and reported lesion at baseline and during follow-up. Imaging based evaluation should always be done rather than clinical examination unless the lesion(s) being followed cannot be imaged but are assessable by clinical exam.

Clinical lesions: Clinical lesions will only be considered measurable when they are superficial and P10 mm diameter as assessed using calipers (e.g. skin nodules). For the case of skin lesions, documentation by color photography including a ruler to estimate the size of the lesion is suggested. As noted above, when lesions can be evaluated by both clinical exam and imaging, imaging evaluation should be undertaken since it is more objective and may also be reviewed at the end of the study.

Chest X-ray: Chest CT is preferred over chest X-ray, particularly when progression is an important endpoint, since CT is more sensitive than X-ray, particularly in identifying new lesions. However, lesions on chest X-ray may be considered measurable if they are clearly defined and surrounded by aerated lung. See Appendix II for more details.

CT, MRI: CT is the best currently available and reproducible method to measure lesions selected for response assessment. This guideline has defined measurability of lesions on CT scan based on the assumption that CT slice thickness is 5 mm or less. As is described in Appendix II, when CT scans have slice thickness greater than 5 mm, the minimum size for a measurable lesion should be twice the slice thickness. MRI is also acceptable in certain situations (e.g. for body scans). More details concerning the use of both CT and MRI for assessment of objective tumour response evaluation are provided in Appendix II.

Ultrasound: Ultrasound is not useful in assessment of lesion size and should not be used as a method of measurement. Ultrasound examinations cannot be reproduced in their entirety for independent review at a later date and, because they are operator dependent, it cannot be guaranteed that the same technique and measurements will be taken from one assessment to the next (described in greater detail in Appendix II). If new lesions are identified by ultrasound in the course of the study, confirmation by CT or MRI is advised. If there is concern about radiation exposure at CT, MRI may be used

instead of CT in selected instances.

Endoscopy, laparoscopy: The utilization of these techniques for objective tumour evaluation is not advised. However, they can be useful to confirm complete pathological response when biopsies are obtained or to determine relapse in trials where recurrence following complete response or surgical resection is an endpoint.

Tumour markers: Tumour markers alone cannot be used to assess objective tumour response. If markers are initially above the upper normal limit, however, they must normalize for a patient to be considered in complete response. Because tumour markers are disease specific, instructions for their measurement should be incorporated into protocols on a disease specific basis. Specific guidelines for both CA-125 response (in recurrent ovarian cancer) and PSA response (in recurrent prostate cancer), have been published.16–18 In addition, the Gynecologic Cancer Intergroup has developed CA125 progression criteria which are to be integrated with objective tumour assessment for use in first-line trials in ovarian cancer.

Cytology, histology: These techniques can be used to differentiate between PR and CR in rare cases if required by protocol (for example, residual lesions in tumour types such as germ cell tumours, where known residual benign tumours can remain). When effusions are known to be a potential adverse effect of treatment (e.g. with certain taxane compounds or angiogenesis inhibitors), the cytological confirmation of the neoplastic origin of any effusion that appears or worsens during treatment can be considered if the measurable tumour has met criteria for response or stable disease in order to differentiate between response (or stable disease) and progressive disease.

4. Tumour response evaluation

4.1. Assessment of overall tumour burden and measurable disease

To assess objective response or future progression, it is necessary to estimate the overall tumour burden at baseline and use this as a comparator for subsequent measurements. Only patients with measurable disease at baseline should be included in protocols where objective tumour response is the primary endpoint. Measurable disease is defined by the presence of at least one measurable lesion (as detailed above in Section 3). In studies where the primary endpoint is tumour progression (either time to progression or proportion with progression at a fixed date), the protocol must specify if entry is restricted to those with measurable disease or whether patients having non-measurable disease only are also eligible.

4.2. Baseline documentation of ‘target’ and ‘non-target’ lesions

When more than one measurable lesion is present at baseline all lesions up to a maximum of five lesions total (and a maximum of two lesions per organ) representative of all involved organs should be identified as target lesions and will be recorded and measured at baseline (this means in instances where patients have only one or two organ sites involved a maximum of two and four lesions respectively will be recorded). For evidence to support the selection of only five target lesions, see analyses on a large prospective database in the article by Bogaerts et al.10.

Target lesions should be selected on the basis of their size (lesions with the longest diameter), be representative of all involved organs, but in addition should be those that lend themselves to reproducible repeated measurements. It may be the case that, on occasion, the largest lesion does not lend itself to reproducible measurement in which circumstance the next largest lesion which can be measured reproducibly should be selected. To illustrate this point, see the example in Fig. 3 of Appendix II.

Lymph nodes merit special mention since they are normal anatomical structures which may be visible by imaging even if not involved by tumour. As noted in Section 3, pathological nodes which are defined as measurable and may be identified as target lesions must meet the criterion of a short axis of P15 mm by CT scan. Only the short axis of these nodes will contribute to the baseline sum. The short axis of the node is the diameter normally used by radiologists to judge if a node is involved by solid tumour. Nodal size is normally reported as two dimensions in the plane in which the image is obtained (for CT scan this is almost always the axial plane; for MRI the plane of acquisition may be axial, sagittal or coronal). The smaller of these measures is the short axis. For example, an abdominal node which is reported as being 20 mm · 30 mm has a short axis of 20 mm and qualifies as a malignant, measurable node. In this example, 20 mm should be recorded as the node measurement (See also the example in Fig. 4 in Appendix II). All other pathological nodes (those with short axis P10 mm but <15 mm) should be considered non-target lesions. Nodes that have a short axis <10 mm are considered non-pathological and should not be recorded or followed.

A sum of the diameters (longest for non-nodal lesions, short axis for nodal lesions) for all target lesions will be calculated and reported as the baseline sum diameters. If lymph nodes are to be included in the sum, then as noted above, only the short axis is added into the sum. The baseline sum diameters will be used as reference to further characterize any objective tumour regression in the measurable dimension of the disease.

All other lesions (or sites of disease) including pathological lymph nodes should be identified as non-target lesions and should also be recorded at baseline. Measurements are not required and these lesions should be followed as ‘present’, ‘absent’, or in rare cases ‘unequivocal progression’ (more details to follow). In addition, it is possible to record multiple nontarget lesions involving the same organ as a single item on the case record form (e.g. ‘multiple enlarged pelvic lymph nodes’ or ‘multiple liver metastases’).

4.3. Response criteria

This section provides the definitions of the criteria used to determine objective tumour response for target lesions.

4.3.1. Evaluation of target lesions

Complete Response (CR): Disappearance of all target lesions. Any pathological lymph nodes (whether target or non-target) must have reduction in short axis to <10 mm.

Partial Response (PR): At least a 30% decrease in the sum of diameters of target lesions, taking as reference the baseline sum diameters.

Progressive Disease (PD): At least a 20% increase in the sum of diameters of target lesions, taking as reference the smallest sum on study (this includes the baseline sum if that is the smallest on study). In addition to the relative increase of 20%, the sum must also demonstrate an absolute increase of at least 5 mm. (Note: the appearance of one or more new lesions is also considered progression).

Stable Disease (SD): Neither sufficient shrinkage to qualify for PR nor sufficient increase to qualify for PD, taking as reference the smallest sum diameters while on study.

4.3.2. Special notes on the assessment of target lesions Lymph nodes. Lymph nodes identified as target lesions should always shave the actual short axis measurement recorded (measured in the same anatomical plane as the baseline examination), even if the nodes regress to below 10 mm on study. This means that when lymph nodes are included as target lesions, the ‘sum’ of lesions may not be zero even if complete response criteria are met, since a normal lymph node is defined as having a short axis of <10 mm. Case report forms or other data collection methods may therefore be designed to have target nodal lesions recorded in a separate section where, in order to qualify for CR, each node must achieve a short axis <10 mm. For PR, SD and PD, the actual short axis measurement of the nodes is to be included in the sum of target lesions.

Target lesions that become ‘too small to measure’. While on study, all lesions (nodal and non-nodal) recorded at baseline should have their actual measurements recorded at each subsequent evaluation, even when very small (e.g. 2 mm). However, sometimes lesions or lymph nodes which are recorded as target lesions at baseline become so faint on CT scan that the radiologist may not feel comfortable assigning an exact measure and may report them as being ‘too small to measure’. When this occurs, it is important that a value be recorded on the case report form. If it is the opinion of the radiologist that the lesion has likely disappeared, the measurement should be recorded as 0 mm. If the lesion is believed to be present and is faintly seen but too small to measure, a default value of 5 mm should be assigned (Note: It is less likely that this rule will be used for lymph nodes since they usually have a definable size when normal and are frequently surrounded by fat such as in the retroperitoneum; however, if a lymph node is believed

to be present and is faintly seen but too small to measure, a default value of 5 mm should be assigned in this circumstance as well). This default value is derived from the 5 mm CT slice thickness (but should not be changed with varying CT slice thickness). The measurement of these lesions is potentially non-reproducible, therefore providing this default value will prevent false responses or progressions based upon measurement error. To reiterate, however, if the radiologist is able to provide an actual measure, that should be recorded, even if it is below 5 mm.

Lesions that split or coalesce on treatment. As noted in Appendix II, when non-nodal lesions 'fragment', the longest diameters of the fragmented portions should be added together to calculate the target lesion sum. Similarly, as lesions coalesce, a plane between them may be maintained that would aid in obtaining maximal diameter measurements of each individual lesion. If the lesions have truly coalesced such that they are no longer separable, the vector of the longest diameter in this instance should be the maximal longest diameter for the 'coalesced lesion'.

4.3.3. Evaluation of non-target lesions

This section provides the definitions of the criteria used to determine the tumour response for the group of non-target lesions. While some non-target lesions may actually be measurable, they need not be measured and instead should be assessed only qualitatively at the time points specified in the protocol.

Complete Response (CR): Disappearance of all non-target lesions and normalization of tumour marker level. All lymph nodes must be non-pathological in size (<10 mm short axis).

Non-CR/Non-PD: Persistence of one or more non-target lesion(s) and/or maintenance of tumour marker level above the normal limits.

Progressive Disease (PD): Unequivocal progression (see comments below) of existing non-target lesions. (Note: the appearance of one or more new lesions is also considered progression).

4.3.4. Special notes on assessment of progression of nontarget disease

The concept of progression of non-target disease requires additional explanation as follows:

When the patient also has measurable disease. In this setting, to achieve 'unequivocal progression' on the basis of the non-target disease, there must be an overall level of substantial worsening in non-target disease such that, even in presence of SD or PR in target disease, the overall tumour burden has increased sufficiently to merit discontinuation of therapy (see examples in Appendix II and further details below). A modest 'increase' in the size of one or more non-target lesions is usually not sufficient to quality for unequivocal progression status. The designation of overall progression solely on the basis of change in non-target disease in the face of SD or PR of target disease will therefore be extremely rare.

When the patient has only non-measurable disease. This circumstance arises in some phase III trials when it is not a criterion of study entry to have measurable disease. The same general concepts apply here as noted above, however, in this instance there is no measurable disease assessment to factor into the interpretation of an increase in non-measurable disease burden. Because worsening in non-target disease cannot be easily quantified (by definition: if all lesions are truly non-measurable) a useful test that can be applied when assessing patients for unequivocal progression is to consider if the increase in overall disease burden based on the change in non-measurable disease is comparable in magnitude to the increase that would be required to declare PD for measurable disease: i.e. an increase in tumour burden representing an additional 73% increase in 'volume' (which is equivalent to a 20% increase diameter in a measurable lesion). Examples include an increase in a pleural effusion from 'trace' to 'large', an increase in lymphangitic disease from localized to widespread, or may be described in protocols as 'sufficient to require a change in therapy'. Some illustrative examples are shown in Figs. 5 and 6 in Appendix II. If 'unequivocal progression' is seen, the patient should be considered to have had overall PD at that point. While it would be ideal to have objective criteria to apply to non-measurable disease, the very nature of that disease makes it impossible to do so, therefore the increase must be substantial.

4.3.5. New lesions

The appearance of new malignant lesions denotes disease progression; therefore, some comments on detection of new lesions are important. There are no specific criteria for the identification of new radiographic lesions; however, the finding of a new lesion should be unequivocal: i.e. not attributable to differences in scanning technique, change in imaging modality or findings thought to represent something other than tumour (for example, some 'new' bone lesions may be simply healing or flare of pre-existing lesions). This is particularly important when the patient's baseline lesions show partial or complete response. For example, necrosis of a liver lesion may be reported on a CT scan report as a 'new' cystic lesion, which it is not.

A lesion identified on a follow-up study in an anatomical location that was not scanned at baseline is considered a new lesion and will indicate disease progression. An example of this is the patient who has visceral disease at baseline and while on study has a CT or MRI brain ordered which reveals metastases. The patient's brain metastases are considered to be evidence of PD even if he/she did not have brain imaging at baseline.

If a new lesion is equivocal, for example because of its small size, continued therapy and follow-up evaluation will clarify if it represents truly new disease. If repeat scans confirm there is definitely a new lesion, then progression should be declared using the date of the initial scan.

While FDG-PET response assessments need additional study, it is sometimes reasonable to incorporate the use of FDG-PET scanning to complement CT scanning in assessment of progression (particularly possible 'new' disease). New lesions on the basis of FDG-PET imaging can be identified according to the following algorithm:

a. Negative FDG-PET at baseline, with a positive FDG-PET at follow-up is a sign of PD based on a new lesion.
b. No FDG-PET at baseline and a positive FDG-PET at follow-up:

If the positive FDG-PET at follow-up corresponds to a new site of disease confirmed by CT, this is PD. If the positive FDG-PET at follow-up is not confirmed as a new site of disease on CT, additional follow-up CT scans are needed to determine if there is truly progression occurring at that site (if so, the date of PD will be the date of the initial abnormal FDG-PET scan). If the positive FDG-PET at follow-up corresponds to a pre-existing site of disease on CT that is not progressing on the basis of the anatomic images, this is not PD.

4.4. Evaluation of best overall response

The best overall response is the best response recorded from the start of the study treatment until the end of treatment taking into account any requirement for confirmation. On occasion a response may not be documented until after the end of therapy so protocols should be clear if post-treatment assessments are to be considered in determination of best overall response. Protocols must specify how any new therapy introduced before progression will affect best response designation. The patient's best overall response assignment will depend on the findings of both target and non-target disease and will also take into consideration the appearance of new lesions. Furthermore, depending on the nature of the study and the protocol requirements, it may also require confirmatory measurement (see Section 4.6). Specifically, in non-randomized trials where response is the primary endpoint, confirmation of PR or CR is needed to deem either one the 'best overall response'. This is described further below.

4.4.1 Time point response

It is assumed that at each protocol specified time point, a response assessment occurs. Table 1 on the next page provides a summary of the overall response status calculation at each time point for patients who have measurable disease at baseline.

When patients have non-measurable (therefore non-target) disease only, Table 2 is to be used.

4.4.2 Missing assessments and unevaluable designation When no imaging/measurement is done at all at a particular time point, the patient is not evaluable (NE) at that time point. If only a subset of lesion measurements is made at an assessment, usually the case is also considered NE at that time point, unless a convincing argument can be made that the contribution of the individual missing lesion(s) would not change the assigned time point response. This would be most likely to happen in the case of PD. For example, if a patient had a baseline sum of 50 mm with three measured

lesions and at follow-up only two lesions were assessed, but those gave a sum of 80 mm, the patient will have achieved PD status, regardless of the contribution of the missing lesion.

4.4.3 Best overall response: all time points

The best overall response is determined once all the data for the patient is known.

Best response determination in trials where confirmation of complete or partial response IS NOT required: Best response in these trials is defined as the best response across all time points (for example, a patient who has SD at first assessment, PR at second assessment, and PD on last assessment has a best overall response of PR). When SD is believed to be best response, it must also meet the protocol specified minimum time from baseline. If the minimum time is not met when SD is otherwise the best time point response, the patient's best response depends on the subsequent assessments. For example, a patient who has SD at first assessment, PD at second and does not meet minimum duration for SD, will have a best response of PD. The same patient lost to follow-up after the first SD assessment would be considered inevaluable.

Best response determination in trials where confirmation of complete or partial response IS required: Complete or partial responses may be claimed only if the criteria for each are met at a subsequent time point as specified in the protocol (generally 4 weeks later). In this circumstance, the best overall response can be interpreted as in Table 3.

4.4.4. Special notes on response assessment

When nodal disease is included in the sum of target lesions and the nodes decrease to 'normal' size (<10 mm), they may still have a measurement reported on scans. This measurement should be recorded even though the nodes are normal in order not to overstate progression should it be based on increase in size of the nodes. As noted earlier, this means that patients with CR may not have a total sum of 'zero' on the case report form (CRF).

In trials where confirmation of response is required, repeated 'NE' time point assessments may complicate best response determination. The analysis plan for the trial must address how missing data/assessments will be addressed in determination of response and progression. For example, in most trials it is reasonable to consider a patient with time point responses of PR-NE-PR as a confirmed response.

Patients with a global deterioration of health status requiring discontinuation of treatment without objective evidence of disease progression at that time should be reported as 'symptomatic deterioration'. Every effort should be made to document objective progression even after discontinuation of treatment. Symptomatic deterioration is not a descriptor of an objective response: it is a reason for stopping study therapy. The objective response status of such patients is to be determined by evaluation of target and non-target disease as shown in Tables 1–3.

Conditions that define 'early progression, early death and inevaluability' are study specific and should be clearly described in each protocol (depending on treatment duration, treatment periodicity).

In some circumstances it may be difficult to distinguish residual disease from normal tissue. When the evaluation of complete response depends upon this determination, it is recommended that the residual lesion be investigated (fine needle aspirate/biopsy) before assigning a status of complete response. FDG-PET may be used to upgrade a response to a CR in a manner similar to a biopsy in cases where a residual radiographic abnormality is thought to represent fibrosis or scarring. The use of FDG-PET in this circumstance should be prospectively described in the protocol and supported by disease specific medical literature for the indication. However, it must be acknowledged that both approaches may lead to false positive CR due to limitations of FDG-PET and biopsy resolution/sensitivity.

For equivocal findings of progression (e.g. very small and uncertain new lesions; cystic changes or necrosis in existing lesions), treatment may continue until the next scheduled assessment. If at the next scheduled assessment, progression is confirmed, the date of progression should be the earlier date when progression was suspected.

4.5. Frequency of tumour re-evaluation

Frequency of tumour re-evaluation while on treatment should be protocol specific and adapted to the type and schedule of treatment. However, in the context of phase II studies where the beneficial effect of therapy is not known, follow-up every 6–8 weeks (timed to coincide with the end of a cycle) is reasonable. Smaller or greater time intervals than these could be justified in specific regimens or circumstances. The protocol should specify which organ sites are to be evaluated at baseline (usually those most likely to be involved with metastatic disease for the tumour type under study) and how often evaluations are repeated. Normally, all target and non-target sites are evaluated at each assessment. In selected circumstances certain non-target organs may be evaluated less frequently. For example, bone scans may need to be repeated only when complete response is identified in target disease or when progression in bone is suspected.

After the end of the treatment, the need for repetitive tumour evaluations depends on whether the trial has as a goal the response rate or the time to an event (progression/death). If 'time to an event' (e.g. time to progression, disease-free survival, progression-free survival) is the main endpoint of the study, then routine scheduled re-evaluation of protocol specified sites of disease is warranted. In randomized comparative trials in particular, the scheduled assessments should be performed as identified on a calendar schedule (for example: every 6–8 weeks on treatment or every 3–4 months after treatment) and should not be affected by delays in therapy, drug holidays or any other events that might lead to imbalance in a treatment arm in the timing of disease assessment.

Table 1 – Time point response: patients with target (+/–non-target) disease.

Target lesions	Non-target lesions	New lesions	Overall response
CR	CR	No	CR
CR	Non-CR/non-PD	No	PR
CR	Not evaluated	No	PR
PR	Non-PD or not all evaluated	No	PR
SD	Non-PD or not all evaluated	No	SD
Not all evaluated	Non-PD	No	NE
PD	Any	Yes or No	PD
Any	PD	Yes or No	PD
Any	Any	Yes	PD

CR = complete response, PR = partial response, SD = stable disease, PD = progressive disease, and NE = inevaluable.

4.6. Confirmatory measurement/duration of response

4.6.1. Confirmation

In non-randomized trials where response is the primary endpoint, confirmation of PR and CR is required to ensure responses identified are not the result of measurement error. This will also permit appropriate interpretation of results in the context of historical data where response has traditionally required confirmation in such trials (see the paper by Bogaerts et al. in this Special Issue10). However, in all other circumstances, i.e. in randomized trials (phase II or III) or studies where stable disease or progression are the primary end points, confirmation of response is not required since it will

Table 2 – Time point response: patients with non-target disease only.

Non-target lesions	New lesions	Overall response
CR	No	CR
Non-CR/non-PD	No	Non-CR/non-PD[a]
Not all evaluated	No	NE
Unequivocal PD	Yes or No	PD
Any	Yes	PD

CR = complete response, PD = progressive disease, and NE = inevaluable.
a 'Non-CR/non-PD' is preferred over 'stable disease' for non-target disease since SD is increasingly used as endpoint for assessment of efficacy in some trials so to assign this category when no lesions can be measured is not advised.

Table 3 – Best overall response when confirmation of CR and PR required.

Overall response First time point	Overall response Subsequent time point	BEST overall response
CR	CR	CR
CR	PR	SD, PD or PR[a]
CR	SD	SD provided minimum criteria for SD duration met, otherwise, PD
CR	PD	SD provided minimum criteria for SD duration met, otherwise, PD
CR	NE	SD provided minimum criteria for SD duration met, otherwise NE
PR	CR	PR
PR	PR	PR
PR	SD	SD
PR	PD	SD provided minimum criteria for SD duration met, otherwise, PD
PR	NE	SD provided minimum criteria for SD duration met, otherwise NE
NE	NE	NE

CR = complete response, PR = partial response, SD = stable disease, PD = progressive disease, and NE = inevaluable.
a If a CR is truly met at first time point, then any disease seen at a subsequent time point, even disease meeting PR criteria relative to baseline,
makes the disease PD at that point (since disease must have reappeared after CR). Best response would depend on whether minimum duration
for SD was met. However, sometimes 'CR' may be claimed when subsequent scans suggest small lesions were likely still present and in fact the
patient had PR, not CR at the first time point. Under these circumstances, the original CR should be changed to PR and the best response is PR.

not add value to the interpretation of trial results. However, elimination of the requirement for response confirmation may increase the importance of central review to protect against bias, in particular in studies which are not blinded.

In the case of SD, measurements must have met the SD criteria at least once after study entry at a minimum interval (in general not less than 6–8 weeks) that is defined in the study protocol.

4.6.2. Duration of overall response

The duration of overall response is measured from the time measurement criteria are first met for CR/PR (whichever is first recorded) until the first date that recurrent or progressive disease is objectively documented (taking as reference for progressive disease the smallest measurements recorded on study).

The duration of overall complete response is measured from the time measurement criteria are first met for CR until the first date that recurrent disease is objectively documented.

4.6.3. Duration of stable disease

Stable disease is measured from the start of the treatment (in randomized trials, from date of randomization) until the criteria for progression are met, taking as reference the smallest sum on study (if the baseline sum is the smallest, this is the reference for calculation of PD).

The clinical relevance of the duration of stable disease varies in different studies and diseases. If the proportion of patients achieving stable disease for a minimum period of time is an endpoint of importance in a particular trial, the protocol should specify the minimal time interval required between two measurements for determination of stable disease.

Note: The duration of response and stable disease as well as the progression-free survival are influenced by the frequency of follow-up after baseline evaluation. It is not in the scope of this guideline to define a standard follow-up frequency. The frequency should take into account many parameters including disease types and stages, treatment periodicity and standard practice. However, these limitations of the precision of the measured endpoint should be taken into account if comparisons between trials are to be made.

4.7. Progression-free survival/proportion progression-free

4.7.1. Phase II trials

This guideline is focused primarily on the use of objective response end points for phase II trials. In some circumstances, 'response rate' may not be the optimal method to assess the potential anticancer activity of new agents/regimens. In such cases 'progression-free survival' (PFS) or the 'proportion progression-free' at landmark time points, might be considered appropriate alternatives to provide an initial signal of biologic effect of new agents. It is clear, however, that in an uncontrolled trial, these measures are subject to criticism since an apparently promising observation may be related to biological factors such as patients' election and not the impact of the intervention. Thus, phase II screening trials utilizing these end points are best designed with a randomized control. Exceptions may exist where the behavior patterns of certain cancers are so consistent (and usually consistently poor), that a non-randomized trial is justifiable (see for example van Glabbeke et al.). However, in these cases it will be essential to document with care the basis for estimating the expected PFS or proportion progression-free in the absence of a treatment effect.

Appendix V: European Organization for Research and Treatment of Cancer Quality of Life Questionnaire – Core 30

EORTC QLQ-C30 (version 3)

We are interested in some things about you and your health. Please answer all of the questions yourself by circling the number that best applies to you. There are no "right" or "wrong" answers. The information that you provide will remain strictly confidential.

Please fill in your initials:
Your birthdate (Day, Month, Year):
Today's date (Day, Month, Year):

	Not at All	A Little	Quite a Bit	Very Much
1. Do you have any trouble doing strenuous activities, like carrying a heavy shopping bag or a suitcase?	1	2	3	4
2. Do you have any trouble taking a long walk?	1	2	3	4
3. Do you have any trouble taking a short walk outside of the house?	1	2	3	4
4. Do you need to stay in bed or a chair during the day?	1	2	3	4
5. Do you need help with eating, dressing, washing yourself or using the toilet?	1	2	3	4

**During the past week:**	Not at All	A Little	Quite a Bit	Very Much
6. Were you limited in doing either your work or other daily activities?	1	2	3	4
7. Were you limited in pursuing your hobbies or other leisure time activities?	1	2	3	4
8. Were you short of breath?	1	2	3	4
9. Have you had pain?	1	2	3	4
10. Did you need to rest?	1	2	3	4
11. Have you had trouble sleeping?	1	2	3	4
12. Have you felt weak?	1	2	3	4
13. Have you lacked appetite?	1	2	3	4
14. Have you felt nauseated?	1	2	3	4
15. Have you vomited?	1	2	3	4
16. Have you been constipated?	1	2	3	4

Please go on to the next page

**During the past week:**	Not at All	A Little	Quite a Bit	Very Much
17. Have you had diarrhea?	1	2	3	4
18. Were you tired?	1	2	3	4
19. Did pain interfere with your daily activities?	1	2	3	4
20. Have you had difficulty in concentrating on things, like reading a newspaper or watching television?	1	2	3	4
21. Did you feel tense?	1	2	3	4
22. Did you worry?	1	2	3	4
23. Did you feel irritable?	1	2	3	4
24. Did you feel depressed?	1	2	3	4
25. Have you had difficulty remembering things?	1	2	3	4
26. Has your physical condition or medical treatment interfered with your family life?	1	2	3	4
27. Has your physical condition or medical treatment interfered with your social activities?	1	2	3	4
28. Has your physical condition or medical treatment caused you financial difficulties?	1	2	3	4

For the following questions please circle the number between 1 and 7 that best applies to you

29. How would you rate your overall health during the past week?

1	2	3	4	5	6	7
Very poor						Excellent

30. How would you rate your overall quality of life during the past week?

1	2	3	4	5	6	7
Very poor						Excellent

© Copyright 1995 EORTC Quality of Life Group. All rights reserved. Version 3.0

Appendix VI：European Organization for Research and Treatment of Cancer Quality of Life Questionnaire – Breast Cancer 23

EORTC QLQ - BR23

Patients sometimes report that they have the following symptoms or problems. Please indicate the extent to which you have experienced these symptoms or problems during the past week.

During the past week:	Not at All	A Little	Quite a Bit	Very Much
31. Did you have a dry mouth?	1	2	3	4
32. Did food and drink taste different than usual?	1	2	3	4
33. Were your eyes painful, irritated or watery?	1	2	3	4
34. Have you lost any hair?	1	2	3	4
35. Answer this question only if you had any hair loss: Were you upset by the loss of your hair?	1	2	3	4
36. Did you feel ill or unwell?	1	2	3	4
37. Did you have hot flushes?	1	2	3	4
38. Did you have headaches?	1	2	3	4
39. Have you felt physically less attractive as a result of your disease or treatment?	1	2	3	4
40. Have you been feeling less feminine as a result of your disease or treatment?	1	2	3	4
41. Did you find it difficult to look at yourself naked?	1	2	3	4
42. Have you been dissatisfied with your body?	1	2	3	4
43. Were you worried about your health in the future?	1	2	3	4

During the past four weeks:	Not at All	A Little	Quite a Bit	Very Much
44. To what extent were you interested in sex?	1	2	3	4
45. To what extent were you sexually active? (with or without intercourse)	1	2	3	4
46. Answer this question only if you have been sexually active: To what extent was sex enjoyable for you?	1	2	3	4

Please go on to the next page

**During the past week:**	**Not at All**	**A Little**	**Quite a Bit**	**Very Much**
47. Did you have any pain in your arm or shoulder?	1	2	3	4
48. Did you have a swollen arm or hand?	1	2	3	4
49. Was it difficult to raise your arm or to move it sideways?	1	2	3	4
50. Have you had any pain in the area of your affected breast?	1	2	3	4
51. Was the area of your affected breast swollen?	1	2	3	4
52. Was the area of your affected breast oversensitive?	1	2	3	4
53. Have you had skin problems on or in the area of your affected breast (e.g., itchy, dry, flaky)?	1	2	3	4

Appendix VII：Scoring method of the QLQ-BR23 and EORTC QLQ-C30(raw score and standard score)

Scales/Items (a BBREVIATION)	Property	Scoring methods (raw score)	Standard score
EORTC QLQ-C30			
Global health status（GHS）	-	（Q29+Q30）/2	[(RS-1)/6] ×100
Physical functioning（PF）	Function	(Q1+Q2+Q3+Q4+Q5)/5	[1-(RS-1)/3]×100
Role functioning（RF）	Function	(Q6+Q7)/2	[1-(RS-1)/3]×100
Emotional functioning（EF）	Function	(Q21+Q22+Q23+Q24)/4	[1-(RS-1)/3]×100
Cognitive functioning（CF）	Function	（Q20+Q25）/2	[1-(RS-1)/3]×100
Social functioning（SF）	Function	（Q26+Q27）/2	[1-(RS-1)/3]×100
Fatigue（FA）	Symptom	（Q10+Q12+Q18）/3	[(RS-1)/3]×100
Nausea and vomiting（NV）	Symptom	(Q14+Q15)/2	[(RS-1)/3]×100
Pain（PA）	Symptom	（Q9+Q19）/2	[(RS-1)/3]×100
Dyspnoea（DY）	Symptom	Q8	[(RS-1)/3]×100
Insomnia（SL）	Symptom	Q11	[(RS-1)/3]×100
Appetite loss（AP）	Symptom	Q13	[(RS-1)/3]×100
cONSTIPATION（CO）	Symptom	Q16	[(RS-1)/3]×100
Diarrhoea（DI）	Symptom	Q17	[(RS-1)/3]×100
Financial difficulties（FI）	Symptom	Q28	[(RS-1)/3]×100
EORTC QLQ-BR23			
Body image（BRBI）	Function	（Q39+Q40+Q41+Q42）/4	[1-(RS-1)/3]×100
Sexual functioning（BRSEF）	Function	(Q44+Q45)/2	[1-(RS-1)/3]×100
Sexual enjoyment（BRSEE）	Function	Q46	[1-(RS-1)/3]×100
Future perspective（BRFU）	Function	Q43	[1-(RS-1)/3]×100
Systemic therapy side effects（BRST）	Symptom	(Q31+Q32+Q33+Q34+Q36+Q37+Q38)/7	[(RS-1)/3]×100
Breast symptoms（BRBS）	Symptom	(Q50+Q51+Q52+Q53)/4	[(RS-1)/3]×100
Ary symptoms（BRAS）	Symptom	(Q47+Q48+Q49)/3	[(RS-1)/3]×100
Upset by hair loss（BRHL）	Symptom	Q35	[(RS-1)/3]×100
