# Supplementary material for: A multicentre single arm phase 2 trial of neoadjuvant pyrotinib and letrozole plus dalpiciclib for triple-positive breast cancer
Source: Nat Commun. 2022 Nov 17;13:7043. doi: 10.1038/s41467-022-34838-w (PMC9672048; doi:10.1038/s41467-022-34838-w)
Supplement: Supplementary file 2 — Reporting Summary [file 41467_2022_34838_MOESM2_ESM.pdf]

nature portfolio

Corresponding author(s): Caigang Liu

Last updated by author(s): Nov 1, 2022

Reporting Summary

Nature Portfolio wishes to improve the reproducibility of the work that we publish. This form provides structure for consistency and transparency in reporting. For further information on Nature Portfolio policies, see our Editorial Policies and the Editorial Policy Checklist.

Please do not complete any field with "not applicable" or n/a. Refer to the help text for what text to use if an item is not relevant to your study.
For final submission: please carefully check your responses for accuracy; you will not be able to make changes later.

Statistics

For all statistical analyses, confirm that the following items are present in the figure legend, table legend, main text, or Methods section.

n/a	Confirmed	
☐	☒	The exact sample size (n) for each experimental group/condition, given as a discrete number and unit of measurement
☐	☒	A statement on whether measurements were taken from distinct samples or whether the same sample was measured repeatedly
☐	☒	The statistical test(s) used AND whether they are one- or two-sided<br>*Only common tests should be described solely by name; describe more complex techniques in the Methods section.*
☐	☒	A description of all covariates tested
☐	☒	A description of any assumptions or corrections, such as tests of normality and adjustment for multiple comparisons
☐	☒	A full description of the statistical parameters including central tendency (e.g. means) or other basic estimates (e.g. regression coefficient) AND variation (e.g. standard deviation) or associated estimates of uncertainty (e.g. confidence intervals)
☐	☒	For null hypothesis testing, the test statistic (e.g. F, t, r) with confidence intervals, effect sizes, degrees of freedom and P value noted<br>*Give P values as exact values whenever suitable.*
☒	☐	For Bayesian analysis, information on the choice of priors and Markov chain Monte Carlo settings
☒	☐	For hierarchical and complex designs, identification of the appropriate level for tests and full reporting of outcomes
☒	☐	Estimates of effect sizes (e.g. Cohen's d, Pearson's r), indicating how they were calculated

Our web collection on statistics for biologists contains articles on many of the points above.

Software and code

Policy information about availability of computer code

Data collection	Excel 2013, SAS (version 9.4).
Data analysis	Statistical analyses were performed using SAS (version 9.4).

For manuscripts utilizing custom algorithms or software that are central to the research but not yet described in published literature, software must be made available to editors and reviewers. We strongly encourage code deposition in a community repository (e.g. GitHub). See the Nature Portfolio guidelines for submitting code & software for further information.

Data

Policy information about availability of data

All manuscripts must include a data availability statement. This statement should provide the following information, where applicable:

- Accession codes, unique identifiers, or web links for publicly available datasets
- A description of any restrictions on data availability
- For clinical datasets or third party data, please ensure that the statement adheres to our policy

The raw clinical and imaging data are protected due to patient privacy laws. The datasets generated and/or analyzed during the current study are available from the corresponding author Caigang Liu on request for 10 years; de-identified clinical data and experimental data are available on request sharing, which will need the approval of the Institutional Ethical Committees. De-identified data will then be transferred to the inquiring investigator over secure file transfer. The study protocol

is available as Supplementary Note 1 in the Supplementary Information. The remaining data are available within the Article, Supplementary Information or Source Data file.

Human research participants

Policy information about studies involving human research participants and Sex and Gender in Research.

Reporting on sex and gender	This study only recruited female breast cancer patients.
Population characteristics	Patients' demographic characteristics are shown in detail in Table 1 of this manuscript.
Recruitment	Patients were offered the possibility of participating in the clinical trial by investigators at routine hospital visits in the five study sites, and some were referred from other seven hospitals into this trial.<br>All patients, who met the criteria were included in this study. There was no potential self-selection bias.<br>The inclusion criteria included: women aged 18-80 years; histologically confirmed stage II-III TPBC; treatment-naïve; Karnofsky score ≥ 70; and adequate bone marrow and organ function. TPBC was defined as HER2-positive (3+ by immunohistochemistry, or 2+ with fluorescence in situ hybridization positivity), ER-positive (more than 10% of tumour cells expressing oestrogen receptor by immunohistochemistry), and PR-positive (at least 1% of tumour cells expressing progesterone receptor by immunohistochemistry) breast cancer.<br>The key exclusion criteria were: bilateral breast cancer, inflammatory breast cancer, or occult breast cancer; other malignancies within five years; serious comorbidities (i.e., uncontrolled hypertension, diabetes mellitus, or active infection); pregnancy, or lactating women. Written informed consent was obtained from all patients.
Ethics oversight	The trial protocol was approved by the Medical Ethics Committees of Shengjing Hospital of China Medical University, Baotou Cancer Hospital, Liaohe Oilfield General Hospital, and Yan'an People's Hospital and the Ethics Committee of Cancer Hospital of Harbin Medical University.<br>The study was performed, according to the principles outlined in the Declaration of Helsinki. Written informed consent was obtained from all patients.

Note that full information on the approval of the study protocol must also be provided in the manuscript.

Field-specific reporting

Please select the one below that is the best fit for your research. If you are not sure, read the appropriate sections before making your selection.

☒ Life sciences ☐ Behavioural & social sciences ☐ Ecological, evolutionary & environmental sciences

For a reference copy of the document with all sections, see nature.com/documents/nr-reporting-summary-flat.pdf

Life sciences study design

All studies must disclose on these points even when the disclosure is negative.

Sample size	We calculated the sample size based on Simon's minimax two-stage design. The null hypothesis of pCR rate was 15% and the alternative hypothesis was 26%, with two-sided α of 0.05 and power of 80%. In the first stage, if 12 or more out of 61 patients achieved pCR, recruitment would proceed for additional 20 patients. If 18 or more out of 81 patients achieved pCR, the study treatment would be deemed worthy of future study.
Data exclusions	No data were excluded.
Replication	The replication was not applicable as this study was a clinical study with the unique patient samples. For the detection of Ki-67 expression, test was done once due to restrict volume of tumor sample. However, the assessments were performed by two independent pathologists.
Randomization	No randomization. This was a single-arm study with no comparator arm.
Blinding	No. This was a single-arm open-label study.

Reporting for specific materials, systems and methods

We require information from authors about some types of materials, experimental systems and methods used in many studies. Here, indicate whether each material, system or method listed is relevant to your study. If you are not sure if a list item applies to your research, read the appropriate section before selecting a response.

Materials & experimental systems

n/a	Involved in the study	
☐	☒	Antibodies
☒	☐	Eukaryotic cell lines
☒	☐	Palaeontology and archaeology
☒	☐	Animals and other organisms
☐	☒	Clinical data
☒	☐	Dual use research of concern

Methods

n/a	Involved in the study	
☒	☐	ChIP-seq
☒	☐	Flow cytometry
☒	☐	MRI-based neuroimaging

Antibodies

Antibodies used

Ki-67 expression was determined by immunohistochemistry using a rabbit monoclonal antibody against Ki67 (clone 30-9; lot H36867; Ventana Medical Systems, Inc., Tucson, AZ, USA)

Validation

All antibodies are commercially available and were only used for applications validated by the manufacturer.

Clinical data

Policy information about clinical studies

All manuscripts should comply with the ICMJE guidelines for publication of clinical research and a completed CONSORT checklist must be included with all submissions.

Clinical trial registration

NCT04486911

Study protocol

The study protocol is available in the Supplementary Information file.

Data collection

This study was an investigator-initiated, multicentre trial in China. Between July 27, 2020 and May 13, 2022, 81 patients were recruited to receive neoadjuvant treatment in Shengjing Hospital of China Medical University, Baotou Cancer Hospital, Liaohe Oilfield General Hospital, Cancer Hospital of Harbin Medical University, and Yan'an People's Hospital. Clinical data were entered into a web-based electronic case report form (CRF) by clinical site staff. The origin of the data was the medical records, charts, and reports available at the clinical sites where patients were treated.

Outcomes

The primary endpoint was the rate of pathological complete response (pCR). The pCR was defined as the disappearance of all invasive tumours in the breast and axillary lymph nodes. Secondary endpoints were the rate of breast pCR (bpCR, defined as disappearance of all invasive tumours in the breast), the proportion of patients with residual cancer burden (RCB)-0 or RCB-I, change in Ki67 levels from baseline to surgery (defined as the percentage of positively staining cells within the invasive margin in the examined area), objective response rate (proportion of patients with complete or partial response per RECIST 1.1) at the end of neoadjuvant treatment before surgery, and safety. Exploratory endpoints were changes in scores of different HRQoL dimensions from baseline to each time point.
The resected breast tissues were evaluated for pathological response and RCB. Ki67 expression was assessed at baseline using biopsied tumour samples and after neoadjuvant therapy using resected tissues. Baseline MRI was performed within 4 weeks prior to the initiation of neoadjuvant therapy. Radiographic response to neoadjuvant therapy was evaluated after the second and the fourth cycles of neoadjuvant therapy, and before surgery.
Adverse events were monitored from the day when the patients provided written informed consent to 28 days after the last neoadjuvant therapy, according to the National Cancer Institute Common Terminology Criteria for Adverse Events version 5.0. Adverse events and tolerability were assessed by scheduled physical examinations, electrocardiogram, laboratory tests, and incidental testing.
Health-related quality of life (HRQoL) was measured using the European Organisation for Research and Treatment of Cancer (EORTC) Quality of Life Questionnaire Core-30 (QLQ-C30; version 3) and Breast Cancer-Specific Module (QLQ-BR23): at baseline, at the end of cycles 2 and 4, and before surgery. The Patient-Reported Outcome (PRO) data were scored according to the EORTC scoring manual. A raw score was calculated as the average of items contributing to a scale and standardized to a range of 0 to 100 points. Higher scores on global health status and functioning subscales indicated better status. Higher scores on symptom subscales indicated worse symptoms.

This checklist template is licensed under a Creative Commons Attribution 4.0 International License, which permits use, sharing, adaptation, distribution and reproduction in any medium or format, as long as you give appropriate credit to the original author(s) and the source, provide a link to the Creative Commons license, and indicate if changes were made. The images or other third party material in this article are included in the article's Creative Commons license, unless indicated otherwise in a credit line to the material. If material is not included in the article's Creative Commons license and your intended use is not permitted by statutory regulation or exceeds the permitted use, you will need to obtain permission directly from the copyright holder. To view a copy of this license, visit http://creativecommons.org/licenses/by/4.0/
